# Supplementary material for: Mapping the colorectal cancer patient journey in Egypt: A qualitative study of diagnosis, treatment, and lifestyle perspectives
Source: PLoS One. 2025 Jul 2;20(7):e0326144. doi: 10.1371/journal.pone.0326144 (PMC12220998; doi:10.1371/journal.pone.0326144)
Supplement: S1 Table — (DOCX) [file pone.0326144.s001.docx]

**Table S1: Participant quotes of perceived barriers**

| **Code** | **Example Quote** |
| --- | --- |
| **Individual Level** |  |
| Lack of Awareness & Neglect of symptoms | *"I kept neglecting my symptoms hoping it would resolve on its own, but my daughter told me I should get myself checked" (male, 59 years).* |
| Embarrassment of colonoscopy | *"I was extremely scared and terrified, and I fainted on the ground before going in. I was afraid of something entering, excuse my language, through my rectum" (female, 58 years).* |
| Fear of colonoscopy pain | *"I kept complaining of rectal bleeding for a whole year as I was afraid to do the colonoscopy because I imagined that it would kill me" (female, 60 years).*  *"What made it easier for me to go through with the procedure quickly was realizing that I felt like I was about to die! I was experiencing severe vomiting, diarrhea, and cramps" (female, 65 years).* |
| Chemotherapy impact on diet and physical activity | *"Through three or four days after the chemotherapy session, I was completely having no appetite. I wanted to vomit, my stomach was hurting, and I was feeling dizzy and faint" (female, 41 years).* |
| Colostomy impact on quality of life | *"Honestly, the colostomy is not pleasant at all. It’s a tough experience because sometimes things happen, like the waste bag bursting or leaking" (male, 61 years).*  *"The colostomy bag feels like a noose around my neck. It’s exhausting—disrupting my sleep, making it hard to sit comfortably, to use the restroom, to do anything"(male, 46 years).* |
| Colostomy impact on diet and physical activity | *"I swear, I feel so hungry and I really want to eat, but I avoid eating because of the colostomy. Even the process of dealing with the colostomy cleaning itself is not pleasant at all" (male, 46 years).*  *"I can’t eat like I used to because of the colostomy bag. It needs to be emptied regularly. For example, if I have to go on a trip from Agami to Raml Station, I might not eat beforehand, thinking the commute could fill the bag, and what if there’s no restroom along the way? This situation can be mentally exhausting and stressful for a person" (male, 45 years).* |
| Job loss due to diagnosis and treatment | *"I used to work at a shop, but after this happened to me, I’ve been sitting at home for about three years without working. I haven’t been able to work at all" (male, 60 years).*  *"I also don’t move around much, and my job used to rely heavily on physical activity. Before, I was out all day and only came home at night to sleep. But now, I’m forced to stay at home" (male, 45 years).* |
| Poverty | *"I commute from Al-Agamy, which is far, coming here to Raml Station, and that has costs. I still need dressing changes for my wound and so on, and all of this costs money" (male, 45 years).* |
| **Interpersonal Level** |  |
| Peers’ fear of colonoscopy | *"I know people who have had it done before (the colonoscopy), but they were scaring me about it. They kept saying that colonoscopy is very difficult" (female, 60 years).*  *"I was afraid of the endoscopy—I’d been scared of it for a long time. I had heard about it, and people said it was intense and difficult, so I was hesitant to go through with it" (female, 65 years).* |
| Fear of peer judgment | *"But also some people might be scared of me and others might be upset" (male, 63 years).* |
| **Health Service Organization** |  |
| Misdiagnosis | *"What made the situation worse was that they kept giving me the wrong medications. They gave me ulcer and stomach inflammation meds, and painkillers, and none of it was helping" (female, 61 years).* |
| Lack of systematic diet and physical activity programs | *"No, no one told me about any specific diet, and they didn’t refer me to anyone—no nutritionist, no physical therapist" (female, 58 years).* |
| lack of comprehensive guidance in the treatment journey | *"Honestly, the hardest thing was that I spent about twenty days feeling like a ghost in the ward. I didn’t know what was wrong with me, and I didn’t know what they were doing to me" (male, 38 years).*  *"They didn’t tell me anything at all...I didn’t find out that I was going in for a colon tumor removal surgery until I was right in front of the operating room" (male, 45 years).* |
| Lack of preparation and Patient Education | *"No, no one explained anything to me about the colonoscopy, and the doctors didn’t provide any details. I wasn’t comfortable with it—it was difficult" (female, 52 years).* |
| **Social Context (Culture/Community)** |  |
| Work: Unsupportive work nature for CRC patients | *"I also don’t move around much, and my job used to rely heavily on physical activity. Before, I was out all day and only came home at night to sleep. But now, I’m forced to stay at home"(male, 45 years).*  *"So, when someone hires me to drive their car, they might worry because I won’t be able to make much money. I’m not making much money myself, and my efforts and work aren’t what they used to be" (male, 63 years).* |
| Work: Unaccepted colostomy in the work environment | *"I used to go out, move around, and earn my daily living. Life was going okay. Now, after the surgery, it’s no longer possible. My work has stopped—it involves a lot of movement." – "Has the stoma affected you?" "Of course, it has—it disabled me" (male, 54 years).*  *"I’m always worried the bag might not be sealed well, and it can leak. I could go to Siwa and work there if I didn’t have this bag and catheter" (male, 38 years).* |
| Beliefs: cultural and religious | *"I really care about hygiene when it comes to this topic. I might even stop praying because of a couple of urine spots. I have these issues recurring often" (male, 63 years).* |
| Health literacy and stigma around colonoscopy | *"An enema and colonoscopy are very difficult, unpleasant, painful, and uncomfortable, and I did not know how to administer the enema myself. They would say, 'I’ll get someone to put it on for you.' But I don’t like that and feel disgusted. It’s such a sensitive area" (male, 61 years).*  *“What scared me was the thought of them inserting something into my rectum, and I don't know what they might do" (female, 60 years).* |
| Cancer stigma | *"They told me… you got that awful, malignant disease. It had spread to my colon and intestines, so they had to remove my colon" (female, 65 years).*  *"At first, they didn’t tell me anything. Then, eventually, they said, you have the malignant disease (male, 60 years).* |
| **Health Policy Level** |  |
| Unclear policies for CRC screening protocols and lack of equipment | *"I went to Al Agamy Hospital, where they gave me a painkiller injection and told me to go to Al Qabbari Hospital for an X-ray because they didn’t have the equipment" (male, 45 years).* |
| Lack of insurance and high costs | *"At Al-Qabbari Hospital, they told me the X-ray would cost around 2000 pounds. I didn’t have the money, so they referred me to El-Miry Hospital" (male, 45 years).*  *“Honestly, in the past, we struggled to get approval for treatment under the state's free healthcare fund. Our financial situation is difficult" (male, 54 years).* |
| Lack of healthcare services in rural areas | *"I came because I have a farm in Siwa and had complained several times over two years about a kidney stone. There’s no healthcare there, so I went to Alexandria and had surgery here" (male, 39 years).* |
