# Supplementary material for: Mapping the colorectal cancer patient journey in Egypt: A qualitative study of diagnosis, treatment, and lifestyle perspectives
Source: PLoS One. 2025 Jul 2;20(7):e0326144. doi: 10.1371/journal.pone.0326144 (PMC12220998; doi:10.1371/journal.pone.0326144)
Supplement: S2 Table — (DOCX) [file pone.0326144.s002.docx]

**Table S2: Participant quotes of perceived facilitators**

| **Code** | ***Example Quote*** |
| --- | --- |
| **Individual Level** |  |
| Faith resilience in accepting diagnosis and treatment | *“I'm not upset because, for me, it's a matter of accepting that only what Allah has decreed will happen. Death is inevitable and normal. I’m prepared for it, and if I were to pass away at any moment, it wouldn’t be due to anything other than what was destined" (male, 38 years).*  *"I am a person of faith, believing in God and destiny. I accept everything that comes from Him, and to this day, I continue to say, 'Alhamdulillah,' thanking Him for everything" (female, 61 years).* |
| Desire to recover | *Q: "Tell me, what made it easier for you to cut out the things you love, like sugar and such?"*  *A: "I want to recover from cancer" (female, 60 years).* |
| Good perception of a healthy diet | *"I mean healthy foods like cottage cheese, low-fat items, vegetables, fruits, fish, and lean meat. And the unhealthy foods are pickles, spicy foods, rich sauces, and fatty foods" (male, 60 years).*  *"Any street food like cheese, falafel, grilled chicken, anything from street vendors is not healthy. Street food is not reliable" (male, 45 years).* |
| History and Good perception of physical activity | *"I love walking. I used to walk a lot—like going from Al-Asafra to Bahri on foot, which is about 17 kilometers. Honestly, it’s amazing. It’s well-known for boosting everything in the body" (male, 63 years).* |
| Walking helped with treatment | *“I was used to walking a lot. So, when I came here, the people who were receiving the same treatment were told to walk, and the doctors also advised me to walk. It helps because I had tumors in my leg, and walking helps me with the treatment" (male, 57 years).* |
| **Interpersonal Level** |  |
| Social support from family and friends | *Currently I live with my family, where my siblings never leave me alone or in need, thank God, and my friends always visit me and keep up with how I’m doing” (male, 39 years).*  *“My family was always by my side. When it was time for the surgery, my husband’s siblings told me, ‘The public hospitals take time to get things done, and the doctor says you need surgery immediately. We’ll handle it for you and don’t worry about the money. We’re your family, and we’re here for you.’ After the surgery, my eldest son’s wife—may God bless her—and my daughter would prepare vegetable soup for me. Even now, they’re still so supportive" (female, 61 years).* |
| Peer patients support | *"The fellow patients with me in the ward are truly well-mannered and respectful" (female, 60 years).* |
| Motivation from survivors | *"I had a relative who had cancer and went to get treated at a hospital in Cairo. Thank God, he got married, had children, and is living a healthy life now" (male, 60 years).* |
| **Health Service Organization** |  |
| Positive doctor-patient relationship | *"Everyone here is respectful, and all the doctors are respectful as well" (male, 68 years).* |
| Doctors recommendations for eating healthy food and being physically active | *"The doctor who performed my surgery came to see me. He advised me to stay active but not to lift anything heavy. He also told me to eat vegetables, fruits, dairy, chicken, meat, fish, and everything" (female, 58 years).* |
| **Social Context (Culture/Community)** |  |
| Religious resilience | *"I’m not upset because, for me, it’s a matter of accepting that only what Allah has decreed will happen" (male, 38 years).* |
| NGO support | *"They told me to do the endoscopy with dye contrast imaging in Smouha through ALEX-CPC for free, and I did it successfully" (female, 41 years).* |
| **Health Policy Level** |  |
| Free healthcare access at university hospitals | *"At the El-Miry Hospital, the doctors examined me and immediately sent me to the operating room" (male, 45 years).* |
| Free chemotherapy at university hospitals | *"I always get the chemotherapy from the hospital for free because it’s not available outside, and if it is, it’s very expensive" (male, 46 years).* |
