# Supplementary material for: Mapping the colorectal cancer patient journey in Egypt: A qualitative study of diagnosis, treatment, and lifestyle perspectives
Source: PLoS One. 2025 Jul 2;20(7):e0326144. doi: 10.1371/journal.pone.0326144 (PMC12220998; doi:10.1371/journal.pone.0326144)
Supplement: S1 File — (DOCX) [file pone.0326144.s003.docx]

**Demographic Questionnaire**

1. **What is your gender?**
   1. Male
   2. Female
2. **What is your birthdate?**

dd/mm/yyyy

1. **What is your weight? (kg)**

**………**

1. **What is your height? (cm)**

**………**

1. **Governorate**
   1. Alexandria
   2. Aswan
   3. Asyut
   4. Bani Suef
   5. Cairo
   6. Dakahlia
   7. Damietta
   8. El-Beheira
   9. Fayoum
   10. Gharbia
   11. Giza
   12. Ismailia
   13. Kafr el-Elsheikh
   14. Luxor
   15. Matrouh
   16. Menoufia
   17. Minya
   18. Noorth Sinai
   19. Port Said
   20. Qalyubia
   21. Qena
   22. Seuz
   23. Sharkia
   24. Sohag
   25. South Sinai
   26. The New Valley
   27. The Red Sea
2. **What is your place of residence?**
   1. Urban
   2. Rural
3. **What is your living Condition?**
   1. Alone
   2. With family
4. **How many family members are in your family?**
   1. 1
   2. 2
   3. 3
   4. 4
   5. 5
   6. More than 5
5. **What is your highest level of education?**
   1. Illiterate
   2. Primary education
   3. Secondary education
   4. High school education
   5. Industrial/Commercial/ Technical Diploma
   6. Bachelor's degree
   7. Master’s/Professional degree
6. **What is your marital status?**
   1. Single
   2. Married
   3. Widowed
   4. Divorced
7. **What is your employment Status?**
   1. Unemployed
   2. Working onsite
   3. Working from home
   4. Disabled
   5. Retired
8. **What is your health insurance status?**
   1. Government health insurance
   2. Private health insurance
   3. Not medically insured
9. **What is your monthly income?**
   1. Not enough
   2. Barely enough
   3. Enough
   4. More than enough
10. **What is your current cancer stage?**
    1. Stage 1
    2. Stage 2
    3. Stage 3
    4. Stage 4 or metastatic cancer
    5. Survivor
