# Supplementary material for: Mapping the colorectal cancer patient journey in Egypt: A qualitative study of diagnosis, treatment, and lifestyle perspectives
Source: PLoS One. 2025 Jul 2;20(7):e0326144. doi: 10.1371/journal.pone.0326144 (PMC12220998; doi:10.1371/journal.pone.0326144)
Supplement: Attachment — Submitted filename: PONE-D-25-14121_suggestions.pdf [file pone.0326144.s005.pdf]

PLOS ONE

Mapping the Colorectal Cancer Patient Journey in Egypt: A Qualitative Study of Diagnosis, Treatment, and Lifestyle Perspectives

--Manuscript Draft--

**Manuscript Number:**	PONE-D-25-14121
**Article Type:**	Research Article
**Full Title:**	Mapping the Colorectal Cancer Patient Journey in Egypt: A Qualitative Study of Diagnosis, Treatment, and Lifestyle Perspectives
**Short Title:**	Colorectal Cancer Journey in Egypt: Diagnosis, Treatment & Lifestyle Insights
**Corresponding Author:**	Mahmoud Bassiony, M.D.<br>Alexandria University Faculty of Medicine<br>Alexandria, Alexandria EGYPT
**Keywords:**	Colorectal cancer, Cancer screening, Patient experiences, Egypt, Qualitative methods, Social-ecological model, Healthy diet and physical activity
**Abstract:**	Background<br>Colorectal cancer (CRC) in Egypt presents a significant public health challenge, ranking as the 7th most common cancer and the 8th leading cause of cancer deaths. Understanding patients' experiences is crucial to inform tailored screening and supportive care. This study aims to explore Egyptian CRC patients' experiences of diagnoses, treatment, and perceptions of a healthy diet and physical activity.<br>Methods<br>Utilizing the Social Ecological Model (SEM) as a theoretical framework, we conducted one-hour, face-to-face, semi-structured interviews with 19 CRC patients in Alexandria, Egypt, between August and September 2023. Audio recorded interviews were transcribed and analyzed thematically to identify patterns across individual, interpersonal, organizational, community, and policy levels of the SEM.<br>Results<br>The participants, 12 males and 7 females with a mean age of 53.4 years, predominantly came from low-income backgrounds, with the majority being unemployed 47.4% or disabled 31.6%. We identified facilitators and barriers to optimal care at several levels of the SEM. The main barriers were as follows: lack of awareness, symptom neglect, fear and embarrassment of colonoscopy, limited diet and activity from chemotherapy and colostomy, job loss, and poverty (individual); peers fear of colonoscopy and judgment (interpersonal); misdiagnosis, inadequate patient education, and lack of structured diet and activity programs (health organization); unsupportive work environments, cultural and religious beliefs, health literacy, and cancer stigma (social context); and unclear screening policies, lack of equipment, lack of insurance, high costs, and limited rural healthcare (policy). The main facilitators were faith resilience and positive perceptions of diet and activity (individual); family and friend support (interpersonal); positive doctor-patient relationships (health organization); NGO support (community); and free healthcare at university hospitals (policy).<br>Conclusion<br>The study findings emphasize the need for improved awareness, education, support systems, enhanced healthcare access, and targeted policy changes, especially in rural areas, to improve diagnosis and treatment outcomes.
**Order of Authors:**	Assem Gebreal
	Karim Abdeltawab
	Omar Hesham
	Samr Kolkas
	Somaia Khamess
	Omnia Fouad
	Mahmoud Ebeid

Powered by Editorial Manager® and ProduXion Manager® from Aries Systems Corporation

	Omar Tarek
	Aya Khaled
	Hamza Mahmoud
	Mahmoud Bassiony, M.D.
	Yara Adel
	Muhammed Helmi
	Maryam Mansour
	Omnia Nahas
	Barbara Hansen, PhD
	Waleed Arafat, MD, PhD
	Mona Fouad, MD, PhD
	Ahmed Ashour Badawy, MD, PhD
	Lori Bateman, PhD
**Opposed Reviewers:**	
**Additional Information:**	
**Question**	**Response**
**Financial Disclosure**<br><br>Enter a financial disclosure statement that describes the sources of funding for the work included in this submission. Review the submission guidelines for detailed requirements. View published research articles from *PLOS ONE* for specific examples.<br><br>This statement is required for submission and **will appear in the published article** if the submission is accepted. Please make sure it is accurate.<br><br>**Funded studies**<br>Enter a statement with the following details:<br>• Initials of the authors who received each award<br>• Grant numbers awarded to each author<br>• The full name of each funder<br>• URL of each funder website<br>• Did the sponsors or funders play any role in the study design, data collection and analysis, decision to publish, or preparation of the manuscript?<br><br>Did you receive funding for this work?	Yes

Please add funding details.<br>as follow-up to "**Financial Disclosure**<br><br>Enter a financial disclosure statement that describes the sources of funding for the work included in this submission. Review the submission guidelines for detailed requirements. View published research articles from *PLOS ONE* for specific examples.<br><br>This statement is required for submission and **will appear in the published article** if the submission is accepted. Please make sure it is accurate.<br><br>**Funded studies**<br>Enter a statement with the following details:<br>• Initials of the authors who received each award<br>• Grant numbers awarded to each author<br>• The full name of each funder<br>• URL of each funder website<br>• Did the sponsors or funders play any role in the study design, data collection and analysis, decision to publish, or preparation of the manuscript?<br><br>Did you receive funding for this work?"	This study was supported by an institutional grant from the UAB Mary Heersink Institute of Global Health at the University of Alabama at Birmingham, Birmingham, AL, USA.
Please select the country of your main research funder (please select carefully as in some cases this is used in fee calculation).<br>as follow-up to "**Financial Disclosure**<br><br>Enter a financial disclosure statement that describes the sources of funding for the work included in this submission. Review the submission guidelines for detailed requirements. View published research articles from *PLOS ONE* for specific examples.<br><br>This statement is required for submission and **will appear in the published article** if the submission is accepted. Please make sure it is accurate.	UNITED STATES - US

**Funded studies**<br>Enter a statement with the following details:<br>• Initials of the authors who received each award<br>• Grant numbers awarded to each author<br>• The full name of each funder<br>• URL of each funder website<br>• Did the sponsors or funders play any role in the study design, data collection and analysis, decision to publish, or preparation of the manuscript?<br><br>Did you receive funding for this work?"	
**Competing Interests**<br><br>Use the instructions below to enter a competing interest statement for this submission. On behalf of all authors, disclose any competing interests that could be perceived to bias this work—acknowledging all financial support and any other relevant financial or non-financial competing interests.<br><br>This statement is required for submission and **will appear in the published article** if the submission is accepted. Please make sure it is accurate and that any funding sources listed in your Funding Information later in the submission form are also declared in your Financial Disclosure statement.<br><br>View published research articles from *PLOS ONE* for specific examples.	The authors have declared that no competing interests exist.

**NO authors have competing interests**<br><br>Enter: *The authors have declared that no competing interests exist.*<br><br>**Authors with competing interests**<br><br>Enter competing interest details beginning with this statement:<br><br>*I have read the journal's policy and the authors of this manuscript have the following competing interests: [insert competing interests here]*<br><br>* typeset	
**Ethics Statement**<br><br>Enter an ethics statement for this submission. This statement is required if the study involved:<br><br>• Human participants<br>• Human specimens or tissue<br>• Vertebrate animals or cephalopods<br>• Vertebrate embryos or tissues<br>• Field research<br><br>Write "N/A" if the submission does not require an ethics statement.<br><br>General guidance is provided below. Consult the submission guidelines for detailed instructions. **Make sure that all information entered here is included in the Methods section of the manuscript.**	The study was conducted in accordance with the Declaration of Helsinki and approved by the Ethics Committee of the Faculty of Medicine, Alexandria University, Egypt (I.R.B. number: 00012098, date of approval 7/07/2023).

Powered by Editorial Manager® and ProduXion Manager® from Aries Systems Corporation

**Format for specific study types**<br><br>**Human Subject Research (involving human participants and/or tissue)**<br>• Give the name of the institutional review board or ethics committee that approved the study<br>• Include the approval number and/or a statement indicating approval of this research<br>• Indicate the form of consent obtained (written/oral) or the reason that consent was not obtained (e.g. the data were analyzed anonymously)<br><br>**Animal Research (involving vertebrate animals, embryos or tissues)**<br>• Provide the name of the Institutional Animal Care and Use Committee (IACUC) or other relevant ethics board that reviewed the study protocol, and indicate whether they approved this research or granted a formal waiver of ethical approval<br>• Include an approval number if one was obtained<br>• If the study involved *non-human primates*, add *additional details* about animal welfare and steps taken to ameliorate suffering<br>• If anesthesia, euthanasia, or any kind of animal sacrifice is part of the study, include briefly which substances and/or methods were applied<br><br>**Field Research**<br><br>Include the following details if this study involves the collection of plant, animal, or other materials from a natural setting:<br>• Field permit number<br>• Name of the institution or relevant body that granted permission	
**Data Availability**<br><br>Authors are required to make all data underlying the findings described fully available, without restriction, and from the time of publication. PLOS allows rare exceptions to address legal and ethical concerns. See the PLOS Data Policy and FAQ for detailed information.	Yes - all data are fully available without restriction

A Data Availability Statement describing where the data can be found is required at submission. Your answers to this question constitute the Data Availability Statement and **will be published in the article**, if accepted.<br><br>**Important:** Stating 'data available on request from the author' is not sufficient. If your data are only available upon request, select 'No' for the first question and explain your exceptional situation in the text box.<br><br>Do the authors confirm that all data underlying the findings described in their manuscript are fully available without restriction?	
**Describe where the data may be found in full sentences. If you are copying our sample text, replace any instances of XXX with the appropriate details.**<br><br>• If the data are **held or will be held in a public repository**, include URLs, accession numbers or DOIs. If this information will only be available after acceptance, indicate this by ticking the box below. For example: *All XXX files are available from the XXX database (accession number(s) XXX, XXX.)*.<br>• If the data are all contained **within the manuscript and/or Supporting Information files**, enter the following: *All relevant data are within the manuscript and its Supporting Information files.*<br>• If neither of these applies but you are able to provide **details of access elsewhere**, with or without limitations, please do so. For example:<br><br>*Data cannot be shared publicly because of [XXX]. Data are available from the XXX Institutional Data Access / Ethics Committee (contact via XXX) for researchers who meet the criteria for access to confidential data.*<br><br>*The data underlying the results presented in the study are available from (include the name of the third party*	The interview records and data used and/or analyzed during the current study are available from the corresponding author upon reasonable request.

and contact information or URL).<br>• This text is appropriate if the data are owned by a third party and authors do not have permission to share the data.<br><br>* typeset	
Additional data availability information:	

Powered by Editorial Manager® and ProduXion Manager® from Aries Systems Corporation

Dear Editor-in-Chief,

We are pleased to submit our manuscript titled "Mapping the Colorectal Cancer Patient Journey in Egypt: A Qualitative Study of Diagnosis, Treatment, and Lifestyle Perspectives" for consideration in PLOS ONE. This study provides a comprehensive exploration of the experiences of colorectal cancer (CRC) patients in Egypt, focusing on their journey from diagnosis to treatment, and their perceptions of healthy diet and physical activity.

Colorectal cancer is a significant public health challenge in Egypt, ranking as the 7th most common cancer and the 8th leading cause of cancer deaths. Despite its high prevalence, there is a lack of qualitative research exploring the lived experiences of CRC patients in this region, particularly regarding the barriers and facilitators they face in accessing care and adopting healthy lifestyle behaviors. Our study addresses this gap by utilizing the Social Ecological Model (SEM) to analyze the multi-level factors influencing CRC patients' experiences, including individual, interpersonal, organizational, community, and policy-level influences.

The findings of this study highlight critical barriers such as lack of awareness, fear of colonoscopy, financial constraints, and healthcare accessibility issues, particularly in rural areas. Conversely, we identified key facilitators, including social support from family and friends, faith resilience, and free healthcare services at university hospitals. These insights underscore the urgent need for culturally adapted policy reforms, improved healthcare communication, and structured support systems to enhance patient outcomes and quality of life.

This research is particularly relevant to PLOS ONE as it aligns with the journal's commitment to publishing rigorous, interdisciplinary research that addresses global health challenges. Our study not only sheds light on the unique challenges faced by CRC patients in a low- and middle-income country (LMIC) context but also provides actionable recommendations for improving care delivery and survivorship outcomes. By addressing the interplay between cultural, social, and systemic factors, this work contributes to the broader discourse on cancer care and survivorship, particularly in underserved populations.

We believe that our findings will be of interest to the journal's diverse readership, including researchers, healthcare providers, and policymakers, as they highlight the need for culturally sensitive approaches to cancer care and survivorship. We are confident that this manuscript will make a valuable contribution to the literature on cancer care and survivorship, particularly in LMICs where such research is scarce.

Thank you for considering our submission. We look forward to the possibility of sharing our work with the readers of PLOS ONE. Please do not hesitate to contact me if you require any additional information or materials.

Sincerely,
Lori Bateman
Assistant Professor
Med - Preventive Medicine
University of Alabama at Birmingham School of Medicine
Birmingham, AL, USA
loribateman@uabmc.edu
+1 (205) 335-7434
Corresponding Author:
Mahmoud Bassiony, MBBCh
Alexandria University, Faculty of Medicine
Email: mahmoudbassiony@outlook.com

Manuscript

Click here to access/download;Manuscript;Qualitative C-CREP - 17-03-2025 MB.docx

Mapping the Colorectal Cancer Patient Journey in Egypt: A Qualitative Study of Diagnosis, Treatment, and Lifestyle Perspectives

Assem Gebreal[1], Karim Abdeltawab[1], Omar Hesham[1], Samr Kolkas[1], Somaia Khamess[1], Omnia Fouad[1], Mahmoud Ebeid[1], Omar Tarek[1], Aya Khaled, Hamza Mahmoud[1], Mahmoud Bassiony[1], Yara Adel[1], Muhammed Helmi[1], Maryam Mansour[1], Omnia Nahas[2], Barbara Hansen[2], Waleed Arafat[1], Mona Fouad[2], Ahmed Ashour Badawy[1], Lori Bateman[2]*

[1]*Alexandria University, Faculty of Medicine, Alexandria, Egypt*

[2]*University of Alabama at Birmingham Heersink School of Medicine, Birmingham, Alabama, U.S.A.*

*Correspondence should be addressed to Mahmoud Bassiony; mahmoudbassiony@outlook.com,

Mahmoud.abdalla1801@alexmed.edu.eg

https://orcid.org/0000-0002-7690-8029

Abstract

Background

Colorectal cancer (CRC) in Egypt presents a significant public health challenge, ranking as the 7th most common cancer and the 8th leading cause of cancer deaths. Understanding patients' experiences is crucial to inform tailored screening and supportive care. This study aims to explore Egyptian CRC patients' experiences of diagnoses, treatment, and perceptions of a healthy diet and physical activity.

Methods

Utilizing the Social Ecological Model (SEM) as a theoretical framework, we conducted one-hour, face-to-face, semi-structured interviews with 19 CRC patients in Alexandria, Egypt, between August and September 2023. Audio recorded interviews were transcribed and analyzed thematically to identify patterns across individual, interpersonal, organizational, community, and policy levels of the SEM.

Results

The participants, 12 males and 7 females with a mean age of 53.4 years, predominantly came from low-income backgrounds, with the majority being unemployed 47.4% or disabled 31.6%. We identified facilitators and barriers to optimal care at several levels of the SEM. The main barriers were as follows: lack of awareness, symptom neglect, fear and embarrassment of colonoscopy, limited diet and activity from chemotherapy and colostomy, job loss, and poverty (individual); peers fear of colonoscopy and judgment (interpersonal); misdiagnosis, inadequate patient education, and lack of structured diet and activity programs (health organization); unsupportive work environments, cultural and religious beliefs, health literacy, and cancer stigma (social context); and unclear screening policies, lack of equipment, lack of insurance, high costs, and limited rural healthcare (policy). The main facilitators were faith resilience and positive perceptions of diet and activity (individual); family and friend support (interpersonal); positive doctor-patient relationships (health organization); NGO support (community); and free healthcare at university hospitals (policy).

[1].

Conclusion

The study findings emphasize the need for improved awareness, education, support systems, enhanced healthcare access, and targeted policy changes, especially in rural areas, to improve diagnosis and treatment outcomes.

Keywords: Colorectal cancer, Cancer screening, Patient experiences, Egypt, Qualitative methods, Social-ecological model, Healthy diet and physical activity

1. Introduction

Colorectal cancer (CRC) remains a major global health challenge, ranking as the third most common cancer worldwide and the second leading cause of cancer deaths, with 1.9 million new cases and 0.9 million fatalities reported in 2020 [1, 2]. This burden is projected to rise to 3.2 million new cases by 2040 [1].

CRC incidence and mortality varies up to 10-fold worldwide[3]. The highest incidence is reported in high-income countries (HICs), including Australia, New Zealand, US Hungary, and Norway. The Western sedentary lifestyle, coupled with rising obesity rates and increased consumption of animal-based foods, alcohol, and tobacco, has been strongly linked to a higher risk of CRC[4, 5]. However, while HICs have experienced stable or declining incidence and mortality rates for CRC in recent years, low- and middle-income countries (LMICs) are witnessing a rising trend, with rates expected to continue increasing significantly over the next few decades [3, 6].

The rising rates of CRC in LMICs are presumably being driven by the adoption of Western lifestyle behaviors along with limited screening, and preventive measures [6]. For instance, the Eastern Mediterranean Region (EMR) has witnessed an increasing incidence of CRC over the last two decades, ranking as the third most common cancer and the fourth leading cause of cancer death in 2022 [7, 8]. However, in contrast to some European countries and USA, CRC survival rates in the EMR remain significantly lower, highlighting disparities in healthcare access, treatment and outcomes [9, 10].

In Egypt, an LMIC located in the EMR, CRC ranks as the 7th most common cancer and the 8th leading cause of cancer deaths, with 5,940 new cases and 3,096 fatalities recorded in 2022 [11]. Particularly, early-onset colorectal cancer (EOCRC), diagnosed below the age of 50, accounts for 42.4% of new cases [12, 13]. In contrast, this figure is only 10% of the new cases in HICs [14, 15]. Further, the majority of CRC cases are diagnosed at a late stage, leading to an overall survival rate of two years [16].

Although remaining a significant health threat in Egypt, CRC is one of the most preventable cancers globally through screening and lifestyle modification [6]. According to a meta-analysis of eleven observational studies, screening methods, particularly colonoscopy, have been associated with a reduction in both CRC incidence and mortality by over 60% [17]. However, despite the availability of the Egyptian CRC screening guidelines, a nationwide study revealed that only 3.2% of the population had adequate awareness of CRC screening and highlighted low compliance toward CRC screening among patients[18].

Lifestyle interventions including regular exercise and healthy eating patterns have been shown to significantly reduce the risk of colorectal cancer [19, 20]. Research has shown that high dietary fiber intake and adequate physical activity can significantly improve quality of life, lower mortality, and improve prognosis for CRC patients [21-23]. In contrast, sedentary behavior of 10 hours or more per day has been linked to a 62% increase in the risk of all-cause mortality among cancer survivors [24]. In Egypt, it is concerning that a significant majority (84.9%) of CRC patients have poor dietary patterns and are malnourished [25]. Additionally, this population reports a high prevalence of sedentary lifestyles [26]. Aly et al reported that 86% of CRC patients did not meet the recommendations from the World Health Organization (WHO) of at least 150 minutes of moderate-to-vigorous-intensity physical activity weekly [27]. In addition to these physical challenges, CRC patients frequently experience psychological distress, including depression, anxiety, stress, which can further impact their quality of life and prognosis [28].

The combination of poor screening compliance and lack of optimal diet and physical activity during treatment is an alarming sign and endorses EOCRC and the low overall survival rate among CRC patients in Egypt.

Assessing patient experiences, along with the barriers and facilitators to diagnosis, treatment, and healthy lifestyle behaviors, is essential for improving outcomes. Additionally, these insights can guide efforts to increase the acceptance and adoption of CRC screening and lifestyle changes, reduce patient distress, support coping with a cancer diagnosis and subsequent treatment, and ultimately lower incidence rates while improving survival[29, 30].

To the best of our knowledge, no prior studies in Egypt have investigated CRC patient's experience with diagnosis, treatment, diet, and physical activity. Therefore, this study aims to investigate CRC patient's experiences with diagnosis, treatment, and perception of diet and physical activity in Egypt, providing insights that could inform strategies to improve care and outcomes for this vulnerable population.

2. Methodology

2.1. Study Design and Setting

The study employed a qualitative research design through individual semi-structured interviews. To ensure confidentiality, interviews were conducted in a private and quiet space within the Alexandria University Faculty of Medicine (AUFM) Oncology Clinic in Alexandria, Egypt. Patients were approached for interviews while waiting for their appointments, and written consent was obtained before the interviews began. The protocol for this study was approved by the AUFM institutional review board.

2.2. Conceptual Framework

This study was situated within the Social Ecological Model (SEM) framework. The SEM emphasizes multiple levels of influence and supports the idea that behaviors both affect and are affected bidirectionally between the spheres [31]. Applying the SEM can provide a comprehensive understanding of the barriers and facilitators facing CRC patients through their journey from diagnosis to survivorship. Furthermore, the SEM helped structure our findings across the various levels of the model, including the individual, interpersonal, community, organization, and policy levels[32].

2.3. Study Participants

The population of interest was Egyptian adults with a prior diagnosis of colorectal cancer (CRC) receiving treatment at the Alexandria University Oncology Clinic. Inclusion criteria included age of at least 30 years old at the time of diagnosis, having cognitive and physical capacity to provide informed consent, and actively participate in the interviews.

Medical students trained as interviewers approached patients waiting for their appointments at the oncology clinic , provided an overview of the study using the Waiting Room Script, and completed an Eligibility Screener with interested patients. Those who met study criteria were provided with an Information Sheet to ensure their understanding of the study. Once the patient verbally consented to participate, a time, date, and location for the interview was chosen based on the participant's preference.

2.4. Recruitment Period

The recruitment of participants took place between August 1, 2023, and September 30, 2023

2.5. Informed Consent Process

Written informed consent was obtained from all participants prior to the interviews. Participants were provided with an Information Sheet detailing the study objectives, procedures, and their rights. Verbal consent was also obtained at the time of initial contact, which was documented by the interviewer and witnessed by a clinic staff member.

2.6. Interview Duration and Process

Individual semi-structured face-to-face interviews, lasting approximately one hour, were conducted with each participant. The interviews, which were audio recorded and translated into Arabic, focused on the participants' CRC journey, including pre-diagnosis, diagnosis, treatment, and their experiences with barriers and facilitators to achieving a healthy lifestyle. The Patient Interview Topic Guide was used to guide the interview process. Additionally, a Demographic Questionnaire was administered by the interviewer during the interview.

2.7. Statistical Analysis

Transcripts were analyzed using thematic analysis with NVIVO 11 to facilitate the coding process[33]. A three-stage process was implemented to ensure rigor in the analysis. First, a team of 4 investigators independently read the interview transcripts to identify codes and potential themes. Second, the investigators discussed and agreed upon coding categories to develop a coding book. Third, the transcripts were re-read and coded by two independent coders according to the agreed-upon coding structure. Meetings were held during the coding process to address any discrepancies and ensure

consistency. Codes were elevated to theses based on recurrence and depth, where the code represents a critical insight central to our research question. Finally, the investigators organized the themes into facilitators and barriers across levels of the SEM, including individual, interpersonal, community, organization, and policy levels.

3. Results

A total of 19 colorectal cancer patients participated in this study. The majority were males (n = 17, 63.2%). The mean age was 53.4 (±10.2) years, and the mean body mass index (BMI) was 21.4 (±6.4). 31.6% (n=6) of patients were living in rural areas. Out of the 13 participants who reported their monthly income, 53.8% (n = 7) indicated that it was barely enough, while 38.5% (n = 5) reported that it was not enough. Most participants had a primary school education 42.1% (n=8) or an Industrial/Commercial/ Technical Diploma 26.3% (n=5). Notably, most participants were unemployed 47.4% (n=9) or disabled 31.6% (n=6), and 100% (n=19) living without health insurance. Most participants (89.5%) were married and 84.2% (n=16) were living with other family members with a mean number of family members per household of 4.5 ±1.7. Notably, 52.6% of participants were diagnosed with stage 3 or 4. Participant characteristics are summarized in Table 1. Our findings, guided by the SEM framework, are categorized into barriers and facilitators affecting CRC diagnosis, treatment, and lifestyle adherence for CRC patients. Each barrier or facilitator was classified to a specific level within SEM[34]. Figure 1 outlines our findings across SEM levels, with key participant quotes highlighted in Tables 2 and 3.

Table 1: Characteristics of study participants (n = 19)

		Num	%
**Age (Mean & SD)**		54.8 ±10.2	
**Sex**			
	Male	12	63.2
	Female	7	36.8
**BMI (Mean & SD)**		21.4 ±6.4	
	Underweight	6	31.6
	Optimum range	10	52.6
	Overweight	3	15.8

**Governorate**			
	Alexandria	11	57.9
	Behiera	7	36.6
	Gharbia	1	5.3
**Place of Residence**			
	Urban	13	68.4
	Rural	6	31.6
**Living Condition**			
	Living alone	3	15.8
	Living with family	16	84.2
**Number of Family Members (Mean & SD)**		4.5 ±1.7	
**Education level**			
	Illiterate	4	21.2
	Primary education	8	42.1
	Secondary education	2	10.6
	Industrial/Commercial/ Technical Diploma	5	26.3
**Marital Status**			
	Married	17	89.5
	Widowed	2	10.5
**Employment status**			
	Unemployed	9	47.4
	Disabled	6	31.6
	Working onsite	3	15.8
	Working from home	1	5.3
**Health Insurance Status**			
	Not medically insured	19	100
**Monthly Income**			
	Barely enough	7	36.8
	Not enough	5	26.3
	Enough	1	5.3
	Not mentioned	6	31.6
**Cancer Stage**			
	Stage 1	2	10.5
	Stage 2	2	10.5
	Stage 3	5	26.3

	Stage 4 or Metastatic cancer	5	26.3
	Not mentioned	4	21.1
	Survivor	1	5.3

BMI; Body mass index

3.1. Perceived barriers

3.1.1. Individual level barriers

At the individual level, lack of awareness was the most common barrier. Most participants had complaints, such as chronic constipation and abdominal pain lasting for more than two years that was neglected until reaching an advanced stage with serious complications. They perceived their symptoms as minor and expected them to resolve on their own, leading to significant delays in seeking medical attention. One participant shared: *“I kept neglecting my symptoms hoping it would resolve on its own, but my daughter told me I should get myself checked.” (Male, 59 years)* When they revisited physicians for reevaluation, the cancer was already in an advanced stage of malignancy. Other patients only sought medical attention when they experienced visible rectal bleeding, while others did not see any physicians until they presented with complete intestinal obstruction.

When physicians suspected malignancy and recommended further investigation, the most commonly reported barrier was fear. Participants discussed different kinds of fears that came up concerning colonoscopy including fear of pain and not knowing what to expect during the procedure, even dying because of it. One participant recounted, *“I kept complaining of rectal bleeding for a whole year as I was afraid to do the colonoscopy because I imagined that it would kill me before my doctor kept seriously advising me to get it done”* (Female, 60 years).

Many patients perceived embarrassment regarding colonoscopy that stopped them from obtaining the procedure. This overall fear and embarrassment of colonoscopy was so severe that one participant fainted before the procedure, “*I was extremely scared and terrified, and I fainted on the ground before going in. I was afraid of something entering, excuse my language, through my rectum." (femlae, 58 years).* Additionally, more than one patient delayed their diagnosis by resisting colonoscopy for a long time, only consenting when they recognized a worsening of their condition. This was a challenging step for some participants as they did it only when they felt they were about to die. One participant shared (Female, 65 year),

> *"What made it easier for me to go through with the procedure quickly was realizing that I felt like I was about to die! I was experiencing severe vomiting, diarrhea, and cramps. So, I decided to do it to find out what was causing my illness."*

Participants also reported poor appetite from chemotherapy, affecting energy levels for physical activity. Some participants also experienced neuropathy in their extremities and back pain, as a side effect of chemotherapy, hindering their ability to engage in physical activities. *"Through three or four days after the chemotherapy session, I was completely having no appetite. I wanted to vomit, my stomach was hurting, and I was feeling dizzy and faint." (Female, 41 years)*

Another significant barrier was coping with a colostomy. Many participants described the experience as profoundly challenging, severely impacting their lives. Recurrent leak accidents caused embarrassment, affected their social interactions, and ultimately diminished their quality of life. One participant recounted:

> *Honestly, the colostomy is not pleasant at all. It's a tough experience because sometimes things happen, like the waste bag bursting or leaking… while you're cleaned up and everything is fine, the bag suddenly leaks. It's really not a good experience at all."*(Male, 61 years)

This fear of possible leaks also negatively impacted their adherence to a healthy diet and physical activity. Participants expressed that they avoided eating well and felt generally restricted in their activities. *"I swear, I feel so hungry and I really want to eat, but I avoid eating because of the colostomy. Even the process of dealing with the colostomy cleaning itself, excuse my language, is not pleasant at all."* (Male, 46 years)

The lack of employment was another significant barrier to diagnosis and treatment. Most of the participants, especially those who worked in physically demanding jobs, could not continue working after surgery and treatment. Employers often did not support individuals in these circumstances, perceiving them as less productive and beneficial to the business. Some participants described the difficulty of this experience. For example, a 60-year-old male stated, "I used to work at a shop, but after this happened to me, I've been sitting at home for about three years without working. I haven't been able to work at all." "What's the reason?" "It's the colostomy bag and the surgery—I'm just not able to work anymore."

Furthermore, poverty emerged as a major barrier for participants, significantly hindering their ability to obtain a timely diagnosis, access CRC treatment, and maintain consistent follow-up care. Many participants highlighted the financial strain caused by the costs of transportation and repeated medical visits for treatments such as wound dressing and medications. One participant explained: *"I commute from Al-Agamy, which is far, coming here to Raml Station, and that has costs. I still need dressing*

changes for my wound and so on, and all of this costs money. So, it has a bit of an impact as well." (Male, 45 years) Another added "Honestly, in the past, we struggled to get approval for treatment under the state's free healthcare fund. Our financial situation is difficult." (Male, 54 Years

3.1.2. Interpersonal level barriers

At the interpersonal level, one female participant narrated that she was afraid of undergoing a colonoscopy, as some people within her social circle warned her of their bad experiences with coloscopy. *"I know people who have had it done before (the colonoscopy), but they were scaring me about it. They kept saying that colonoscopy is very difficult."* (Female,60 years)

Additionally, another participant observed that individuals in his near circle might become apprehensive or frustrated with his health condition, particularly in the context of managing a colostomy that demands specialized care. This perception contributed to his anxiety and depression, which, in turn, limited his social interactions and heightened his fear of judgment from others. *" But also some people might be scared of me and others might be upset."* (Male, 63 year)

3.1.3. Health service organization barriers

At this level, many participants were initially misdiagnosed, which delayed their treatment and led to complications. Many physicians failed to suspect malignancy during CRC's early presentation. Participants mentioned receiving diagnoses and treatment for diseases like peptic ulcers and gastritis and receiving emollients until the appearance of complications such as rectal bleeding and severe weight loss.

One participant recounted: *"What made the situation worse was that they kept giving me the wrong medications. They gave me ulcer and stomach inflammation meds, and painkillers, and none of it was helping. Eventually, I saw another doctor, and when the issue didn't improve, I ended up not having any bowel movements for about a week and was in severe pain."* (Female, 61 Years)

Another barrier was challenges in procedure preparation. Some patients reported a negative history of colonoscopy, often due to insufficient explanation from healthcare providers, resulting in painful and traumatic experiences that discouraged future medical procedures. A participant shared, *"No, no one explained anything to me about the colonoscopy, and the doctors didn't provide any details. I wasn't comfortable with it—it was difficult." (female, 52)*

Moreover, although physicians advised patients to eat vegetables and a balanced diet and avoid oily fatty food post-surgery, none of the patients received a detailed tailored dietary plan or were referred to a nutritionist. This observation was also reported for physical activity, as some patients were advised

to stay active in general without specific guidance on types of exercises or duration, or were referred to a nutritionist, while others did not receive any recommendation at all.

Moreover, while physicians advised patients to consume vegetables, maintain a balanced diet, and avoid oily or fatty foods after surgery, none of the patients received a detailed, tailored dietary plan or were referred to a nutritionist. Similarly, recommendations for physical activity were inconsistent. Some patients were advised to "stay active" in general but were not provided with specific guidance on the types of exercises, duration, or intensity. Others received no recommendations at all regarding physical activity. Furthermore, none of the patients were referred to a physiotherapist for specialized post-surgical rehabilitation.

Another significant barrier was the lack of comprehensive guidance throughout the treatment journey or consultation. Patients complained about not receiving a satisfactory explanation from healthcare providers about their health conditions, the rationale of medical recommendations, and expected outcomes, especially for those receiving a colostomy, as it hinders many peoples' general activities and work. A participant shared, *"Honestly, the hardest thing was that I spent about twenty days feeling like a ghost in the ward. I didn't know what was wrong with me, and I didn't know what they were doing to me. They would come in, and I was the only one in the middle of the ward."* (Male, 38 years)

Another added: *"They didn't tell me anything at all...I didn't find out that I was going in for a colon tumor removal surgery until I was right in front of the operating room. I had to sign the consent form with my fingerprints" (Male, 45Years)*

3.1.4. ***Social context barriers (culture/community)***

At this level, the lack of workplace accommodations, stigma around visible health conditions, and insufficient support systems exacerbate challenges for individuals with stomas. Several participants discussed how having a stoma significantly impacts their ability to find and maintain employment. They highlighted difficulties managing the waste collection bag during work, including frequent changes and the risk of leaks, which create practical challenges and contribute to feelings of embarrassment and fear of workplace rejection. For example, a 38-year-old male patient shared, "I'm always worried the bag might not be sealed well and it can leak. I could go to Siwa and work there if I didn't have this bag and catheter. These things, if they weren't there, I wouldn't have any problems."

Cultural values and religious beliefs were also perceived as barriers to diagnosis and treatment services. For instance, a 63-year-old male participant reported that using a stoma collection bag was challenging while adhering to religious practices like praying, as any waste leakage could spoil his praying. *"I really care about hygiene when it comes to this topic. I might even stop praying because of a couple of urine spots. I have these issues recurring often."*(E04)

Furthermore, some participants highlighted the community health literacy and stigma surrounding colonoscopy, particularly due to the need for anal penetration, which was perceived as invasive and culturally sensitive. Privacy concerns, cultural norms, and lack of accurate information also influenced participants' perceptions of the colonoscopy process. A participant explained, *"An enema and colonoscopy are very difficult, unpleasant, painful, and uncomfortable and I did not know how to administer the enema myself. They would say, 'I'll get someone to put it on for you.' But I don't like that and feel disgusted. It's such a sensitive area"* (Male, 61 years). Another participant added "What scared me was the thought of them inserting something into my rectum, and I don't know what they might do" (Female, 60 years)

There was also a notable stigma toward cancer within their communities. Therefore, some participants avoided mentioning cancer by its name, referring to it instead as "the malignant disease." Moreover, some caregivers did not inform their loved ones about their cancer diagnosis until the advanced stages of treatment. This delay was due to fears of the psychological impact, stigma, and the pervasive perception that a cancer diagnosis is a curse or death sentence. One participant shared, *"They told me… you got that awful, malignant disease. It had spread to my colon and intestines, so they had to remove my colon."* (Female, 65 year)Another added: *"At first, they didn't tell me anything. Then, eventually, they said, you have the malignant disease (Male, 60 years)."*

3.1.5. Health policy barriers

Patients reported barriers that hinder receiving a definitive diagnosis and treatment at this level due to systemic health policy issues. One of the main barriers reported was the delay caused by multiple referrals between hospitals, often due to unclear policies for CRC screening protocols and a lack of imaging equipment. Another critical barrier was the lack of health insurance, which prevented many patients from affording costly diagnostic procedures in private healthcare facilities. Most of the patients were finally referred to Alexandria's main university hospital at an advanced stage with serious complications. One participant shared,

"I consulted several doctors, and they told me it was irritable bowel syndrome. The last time I felt very ill... I went to Al Agamy Hospital, where they gave me a painkiller injection and told me to go to Al Qabbari Hospital for an X-ray because they didn't have the equipment.At Al-Qabbari Hospital, they told me the X-ray would cost around 2000 pounds. I didn't have the money, so they referred me to El-Miry Hospital." (Male, 45Years)

In addition, several participants reported difficulties in accessing healthcare services, especially those living in rural areas, leading to delayed diagnosis and treatment. Long wait times and the necessity for multiple referrals compounded these delays. Another participant from a rural area mentioned: *"I came*

because I have a farm in Siwa and had complained several times over two years about a kidney stone. There’s no healthcare there, so I went to Alexandria and had surgery here." (Male, 39 years)

3.2. Perceived facilitators

3.2.1. Individual facilitators

Most participants showed resilience in accepting diagnosis and treatment due to their faith in their destiny. One participant shared "I am a person of faith, believing in God and destiny. I accept everything that comes from Him, and to this day, I continue to say, 'Alhamdulillah,' thanking Him for everything." (female, 61 years). This spiritual conviction provided them with strength through their journey, fostering a belief that they were guided and protected by a higher power. Another participant added*“I'm not upset because, for me, it's a matter of accepting that only what Allah has decreed will happen. Death is inevitable and normal. I’m prepared for it, and if I were to pass away at any moment, it wouldn’t be due to anything other than what was destined."* (Male, 38 years)

Participants had a clear desire to recover and regain their health. Many highlighted their efforts to adhere to treatment instructions and make lifestyle changes. One participant shared

> *Q: "Tell me, what made it easier for you to cut out the things you love, like sugar and such?"*
>
> *A: "I want to recover from cancer."* (Female, 60 years)

Although all participants did not have a scientific background on how to make a balanced diet, the majority perceived a healthy diet as one that makes their bodies feel good after consumption. Some participants mentioned that spicy food, fatty or greasy food, and a lot of sugar are bad for health. If a particular food induces feelings of sickness or discomfort, it is considered unhealthy, prompting them to avoid it. Their understanding of a healthy diet generally includes a balanced combination of proteins, carbohydrates, and vegetables.

"I mean healthy foods like cottage cheese, low-fat items, vegetables, fruits, fish, and lean meat And the unhealthy foods are pickles, spicy foods, rich sauces, and fatty foods." Said a 60 year old male participant

Most of the participants mentioned that they avoid junk food and eat only homemade. One participant narrated that *"Any street food like cheese, falafel, grilled chicken, anything from street vendors is not healthy. Street food is not reliable."* (Male, 45Years)

For physical activity, participants perceive it as the ability to carry out daily tasks such as going to work, buying dinner, meeting with friends and family, and navigating through the day. Walking was the only explicitly mentioned form of exercise. One participant reflected, "I love walking. I used to walk a lot—

like going from Al-Asafra to Bahri on foot, which is about 17 kilometers. Honestly, it's amazing. It's well-known for boosting everything in the body." (Male, 63 year)

Additionally, some participants mentioned that being physically active and committing to daily walking helped them go through their therapy sessions and made them feel better in comparison to other patients who are physically inactive.

> *"I was used to walking a lot. So, when I came here, the people who were receiving the same treatment were told to walk, and the doctors also advised me to walk. It helps because I had tumors in my leg, and walking helps me with the treatment." (Male, 57 Years)*

3.2.2. Interpersonal facilitators

Social support was considered an important facilitator. Family members, neighbors, and friends were highly supportive in terms of both financial and psychological support during the treatment journey. Participants narrated that their families helped them prepare meals, ensuring their adherence to doctors' recommendations. *"Currently I live with my family, where my siblings never leave me alone or in need, thank God, and my friends always visit me and keep up with how I'm doing."* (Male, 39 years). Another participant added ""My family was always by my side. When it was time for the surgery, my husband's siblings told me, 'The public hospitals take time to get things done, and the doctor says you need surgery immediately. We'll handle it for you and don't worry about the money'. We're your family, and we're here for you.' After the surgery, my eldest son's wife—may God bless her—and my daughter would prepare vegetable soup for me. Even now, they're still so supportive. Whenever I need anything, they're always there for me."(female, 61 years)

Furthermore, during chemotherapy sessions and other medical procedures at the hospital, fellow patients were cooperative and supported each other, offering encouragement and solidarity. *"The fellow patients with me in the ward are truly well-mannered and respectful."* (Female, 60 years)

Additionally, several participants mentioned that knowing a cancer survivor, especially a family member or close friend, was highly motivating them to engage in the treatment effectively. One participant shared *"I had a relative who had cancer and went to get treated at a hospital in Cairo. Thank God, he got married, had children, and is living a healthy life now. Everything is in God's hands and according to His will."* (*Male, 60 years*)

3.2.3. Health service organization facilitators

At this level, healthcare professionals including physicians and nurses left favorable feedback on participants in terms of dedication, support, empathy, and having good doctor-patient relationships. Participants acknowledged the effort provided by medical professionals, which significantly contributed to their overall treatment experience and emotional well-being. One participant shared *"Everyone here is respectful, and all the doctors are respectful as well." (male, 68 years)*

Healthcare professionals also played a vital role in encouraging patients to maintain a healthy diet and stay physically active. Most patients received guidance during treatment, emphasizing the importance of staying active and discouraging prolonged bed rest. Doctors encouraged walking, even within their homes if they could not go outside. Additionally, they promoted the inclusion of more vegetables in their diet while advising against fatty meals and spicy foods, emphasizing their importance in maintaining proper bowel movement. One participant explained *"The doctor who performed my surgery came to see me. He advised me to stay active but not to lift anything heavy. He also told me to eat vegetables, fruits, dairy, chicken, meat, fish, and everything." (femlae, 58 years)*

3.2.4. ***Social context facilitators(culture/community)***

The resilience demonstrated by participants is deeply influenced by the community aspects of religion. Religious teachings and practices within the community emphasize acceptance of destiny, trust in divine will, and gratitude in the face of adversity. For instance, the frequent use of phrases like "Alhamdulillah" (praise be to God) reflects a broader cultural and religious context that encourages gratitude and acceptance. This communal environment fosters and reinforces the individual beliefs that enable participants to cope with their diagnosis and treatment journey.

Additionally, several participants highlighted the role of NGOs and charity organizations in supporting their treatment. The Alexandria Society of Cancer Patient Care (ALEX-CPC), a registered NGO affiliated with Alexandria University's Clinical Oncology Department, provides free cancer treatments not covered by the government and financial aid for patients who lost their jobs due to cancer. One participant narrated *"They told me to do the endoscopy with dye contrast imaging in Smouha through ALEX-CPC for free, and I did it successfully." (Female, 41 years).*

3.2.5. Health policy facilitators

One of the main facilitators at this level was the seamless and free access to healthcare and treatment at university hospitals. Patients were admitted for surgery during the same week they presented to the ER, with some even undergoing the operation on the very next day. Tumor samples were taken in the operating room (OR), eliminating the need for a separate colonoscopy session. *"At the El-Miry Hospital, the doctors examined me and immediately sent me to the operating room."* (Male, 45 years)

Notably, none of the participants had health insurance. However, the free access to medications and chemotherapy was a smooth process with costs covered at university hospitals due to the health policy, which was crucial for improvement considering the social status and work status of patients. *"I always get the chemotherapy from the hospital for free because it's not available outside, and if it is, it's very expensive."* (Male, 46 years)

Table 2: Participant quotes of perceived barriers

Code	Example Quote
**Individual Level**	
Lack of Awareness & Neglect of symptoms	*"I kept neglecting my symptoms hoping it would resolve on its own, but my daughter told me I should get myself checked."* (Male, 59 years) (E03)
Embarrassment of colonoscopy	*"I was extremely scared and terrified, and I fainted on the ground before going in. I was afraid of something entering, excuse my language, through my rectum."* (Female, 58 years).
Fear of colonoscopy pain	*"I kept complaining of rectal bleeding for a whole year as I was afraid to do the colonoscopy because I imagined that it would kill me."* (Female, 60 years)<br><br>*"What made it easier for me to go through with the procedure quickly was realizing that I felt like I was about to die! I was experiencing severe vomiting, diarrhea, and cramps."* (Female, 65 years)
Chemotherapy impact on diet and physical activity	*"Through three or four days after the chemotherapy session, I was completely having no appetite. I wanted to vomit, my stomach was hurting, and I was feeling dizzy and faint."* (Female, 41 years)
Colostomy impact on quality of life	*"Honestly, the colostomy is not pleasant at all. It's a tough experience because sometimes things happen, like the waste bag bursting or leaking."* (Male, 61 years)<br><br>"The colostomy bag feels like a noose around my neck. It's exhausting—disrupting my sleep, making it hard to sit comfortably, to use the restroom, to do anything."(Male, 46 years)

Colostomy impact on diet and physical activity	*"I swear, I feel so hungry and I really want to eat, but I avoid eating because of the colostomy. Even the process of dealing with the colostomy cleaning itself is not pleasant at all."* (Male, 46 years)<br><br>"I can't eat like I used to because of the colostomy bag. It needs to be emptied regularly. For example, if I have to go on a trip from Agami to Raml Station, I might not eat beforehand, thinking the commute could fill the bag, and what if there's no restroom along the way? This situation can be mentally exhausting and stressful for a person." (Male, 45 years)
Job loss due to diagnosis and treatment	*"I used to work at a shop, but after this happened to me, I've been sitting at home for about three years without working. I haven't been able to work at all."* (Male, 60 years)<br><br>"I also don't move around much, and my job used to rely heavily on physical activity. Before, I was out all day and only came home at night to sleep. But now, I'm forced to stay at home." (Male, 45 years)
Poverty	*"I commute from Al-Agamy, which is far, coming here to Raml Station, and that has costs. I still need dressing changes for my wound and so on, and all of this costs money."* (Male, 45 years)
**Interpersonal Level**	
Peers' fear of colonoscopy	*"I know people who have had it done before (the colonoscopy), but they were scaring me about it. They kept saying that colonoscopy is very difficult."* (Female, 60 years)<br><br>"I was afraid of the endoscopy—I'd been scared of it for a long time. I had heard about it, and people said it was intense and difficult, so I was hesitant to go through with it." (Female, 65 years)
Fear of peer judgment	*"But also some people might be scared of me and others might be upset."* (Male, 63 years)
**Health Service Organization**	
Misdiagnosis	*"What made the situation worse was that they kept giving me the wrong medications. They gave me ulcer and stomach inflammation*

	meds, and painkillers, and none of it was helping." (Female, 61 years)
Lack of systematic diet and physical activity programs	"No, no one told me about any specific diet and they didn't refer me to anyone—no nutritionist, no physical therapist." (femlae, 58 years)
lack of comprehensive guidance in the treatment journey	*"Honestly, the hardest thing was that I spent about twenty days feeling like a ghost in the ward. I didn't know what was wrong with me, and I didn't know what they were doing to me."* (Male, 38 years)<br><br>*"They didn't tell me anything at all...I didn't find out that I was going in for a colon tumor removal surgery until I was right in front of the operating room."* (Male, 45 years)
Lack of preparation and Patient Education	*"No, no one explained anything to me about the colonoscopy, and the doctors didn't provide any details. I wasn't comfortable with it—it was difficult."* (Female, 52 years)
**Social Context (Culture/Community)**	
Work: Unsupportive work nature for CRC patients	"I also don't move around much, and my job used to rely heavily on physical activity. Before, I was out all day and only came home at night to sleep. But now, I'm forced to stay at home." (Male, 45 years)<br><br>*"So, when someone hires me to drive their car, they might worry because I won't be able to make much money. I'm not making much money myself, and my efforts and work aren't what they used to be."* (Male, 63 year)
Work: Unaccepted colostomy in the work environment	"I used to go out, move around, and earn my daily living. Life was going okay. Now after the surgery, it's no longer possible. My work has stopped—it involves a lot of movement." – "Has the stoma affected you?" "Of course, it has—it disabled me." (Male, 54 Years)<br><br>*"I'm always worried the bag might not be sealed well and it can leak. I could go to Siwa and work there if I didn't have this bag and catheter."* (Male, 38 years)

Beliefs: cultural and religious	*"I really care about hygiene when it comes to this topic. I might even stop praying because of a couple of urine spots. I have these issues recurring often."* (Male, 63 years)
Health literacy and stigma around colonoscopy	*"An enema and colonoscopy are very difficult, unpleasant, painful, and uncomfortable and I did not know how to administer the enema myself.*<br>They would say, 'I'll get someone to put it on for you.' But I don't like that and feel disgusted. It's such a sensitive area" (Male, 61 years)<br><br>*"What scared me was the thought of them inserting something into my rectum, and I don't know what they might do"* (Female, 60 years)
Cancer stigma	*"They told me… you got that awful, malignant disease. It had spread to my colon and intestines, so they had to remove my colon."* (Female, 65 years)<br><br>*"At first, they didn't tell me anything. Then, eventually, they said, you have the malignant disease (Male, 60 years)*
**Health Policy Level**	
Unclear policies for CRC screening protocols and lack of equipment	*"I went to Al Agamy Hospital, where they gave me a painkiller injection and told me to go to Al Qabbari Hospital for an X-ray because they didn't have the equipment."* (Male, 45 years)
Lack of insurance and high costs	*"At Al-Qabbari Hospital, they told me the X-ray would cost around 2000 pounds. I didn't have the money, so they referred me to El-Miry Hospital."* (Male, 45 years)<br><br>*"Honestly, in the past, we struggled to get approval for treatment under the state's free healthcare fund. Our financial situation is difficult."* (Male, 54 years)
Lack of healthcare services in rural areas	*"I came because I have a farm in Siwa and had complained several times over two years about a kidney stone. There's no healthcare there, so I went to Alexandria and had surgery here."* (Male, 39 years)

Table 3: Participant quotes of perceived facilitators

Code	Example Quote
**Code**	**Example Quote**
**Individual Level**	
Faith resilience in accepting diagnosis and treatment	*“I'm not upset because, for me, it's a matter of accepting that only what Allah has decreed will happen. Death is inevitable and normal. I’m prepared for it, and if I were to pass away at any moment, it wouldn’t be due to anything other than what was destined."* (Male, 38 years)<br><br>*"I am a person of faith, believing in God and destiny. I accept everything that comes from Him, and to this day, I continue to say, 'Alhamdulillah,' thanking Him for everything."* (Female, 61 years)
Desire to recover	*"I want to recover from cancer."* (Female, 60 years)
Good perception of a healthy diet	*"I mean healthy foods like cottage cheese, low-fat items, vegetables, fruits, fish, and lean meat. And the unhealthy foods are pickles, spicy foods, rich sauces, and fatty foods."* (Male, 60 years)<br><br>*"Any street food like cheese, falafel, grilled chicken, anything from street vendors is not healthy. Street food is not reliable." (Male, 45Years)*
History and Good perception of physical activity	*"I love walking. I used to walk a lot—like going from Al-Asafra to Bahri on foot, which is about 17 kilometers. Honestly, it’s amazing. It’s well-known for boosting everything in the body."* (Male, 63 years)
Walking helped with treatment	*“I was used to walking a lot. So, when I came here, the people who were receiving the same treatment were told to walk, and the doctors also advised me to walk. It helps because I had tumors in my leg, and walking helps me with the treatment." (Male, 57 Years)*
**Interpersonal Level**	
Social support from family and friends	*Currently I live with my family, where my siblings never leave me alone or in need, thank God, and my friends always visit me and keep up with how I’m doing.”* (Male, 39 years).<br><br>*"My family was always by my side. When it was time for the surgery, my husband’s siblings told me, ‘We’ll handle it for you and don’t worry about the money.’"* (Female, 61 years).

Peer patients support	*"The fellow patients with me in the ward are truly well-mannered and respectful."* (Female, 60 years).
Motivation from survivors	*"I had a relative who had cancer and went to get treated at a hospital in Cairo. Thank God, he got married, had children, and is living a healthy life now."* (Male, 60 years).
**Health Service Organization**	
Positive doctor-patient relationship	*"Everyone here is respectful, and all the doctors are respectful as well."* (Male, 68 years).
Doctors recommendations for eating healthy food and being physically active	*"The doctor who performed my surgery came to see me. He advised me to stay active but not to lift anything heavy. He also told me to eat vegetables, fruits, dairy, chicken, meat, fish, and everything."* (Female, 58 years).
**Social Context (Culture/Community)**	
Religious resilience	*"I'm not upset because, for me, it's a matter of accepting that only what Allah has decreed will happen."* (Male, 38 years).
NGO support	*"They told me to do the endoscopy with dye contrast imaging in Smouha through ALEX-CPC for free, and I did it successfully."* (Female, 41 years).
**Health Policy Level**	
Free healthcare access at university hospitals	*"At the El-Miry Hospital, the doctors examined me and immediately sent me to the operating room."* (Male, 45 years).
Free chemotherapy at university hospitals	*"I always get the chemotherapy from the hospital for free because it's not available outside, and if it is, it's very expensive."* (Male, 46 years)

4. Discussion:

This study provides a comprehensive exploration of CRC patients' journeys toward achieving a healthy lifestyle and accessing effective care in Egypt. Using the SEM, we identified barriers and facilitators faced by each patient's individual experience across the individual, interpersonal, organizational, community, and policy levels. The thematic analysis revealed a complex interconnection between systemic challenges, cultural influences, and individual behaviors, offering insights for developing targeted interventions to support CRC patients in Egypt.

One key finding from the diagnosis phase is that more than half of our participants presented with intestinal obstruction in the emergency room (ER) at advanced cancer stage 3 or 4 with metastases. This strongly demonstrates delayed diagnoses of CRC among patients in Egypt. Different studies reported an estimated survival being 26 months for stage 2 and a median overall survival being 2 years [35, 36]. The reasons for this phenomenon span the SEM levels.

Per health policy in Egyptian clinics, there is a lack of applying clear screening protocols or guidelines for early detection of CRC, particularly in rural areas, where healthcare services are scarce. Developing a healthcare policy to provide facilities and health services in rural areas is essential for timely diagnosis and treatment[37]. This could involve setting up more clinics, mobile healthcare units, and telemedicine services to ensure timely diagnosis and treatment. Furthermore, there were challenges in obtaining healthcare services and investigation due to high costs in private sectors and lack of equipment. These findings align with existing literature that individuals from LMICs face substantial cost and accessibility barriers to screening, resulting in late-stage diagnoses and poorer outcomes [38].

Length of time between referrals and diagnosis typically contribute to late-stage CRC, identified tertiary care hospitals. Despite availability of screening guidelines, evidence points to a lack of routine screening in current practice in Egypt. For instance, in a nationwide survey, only 2.3% of eligible participants were referred for CRC screening[18]. Furthermore, Bateman et al. found that many Egyptian physicians reported screening only high-risk or symptomatic patients [39].

In contrast, free treatment policies at tertiary care educational hospitals were highlighted as a crucial facilitator. It provided access to urgent surgeries, chemotherapy, and oncology care funded by the government for uninsured patients. This quick transition allowed for timely consultations with oncologists and initiation of treatment compared with longer intervals in developed countries [40-42]. Moreover, the participants received treatment supported by government funds, indicating the accessibility and affordability of cancer care in Egypt. This is especially significant in Egypt, where the notably high poverty rate is at 29.7% and the main healthcare financing source is out-of-pocket (OOP) expenditure, representing more than 60% of total health expenditure [43, 44].

This finding highlights the importance of government support in ensuring equitable access to cancer treatment.

As evidenced in our study, unsupportive work environments, low health literacy, cultural and religious beliefs, and cancer stigma present challenges for most Egyptians. Employment types that require high physical demands are often unsuitable for cancer patients and those with colostomies, further complicating their ability to maintain a stable income. Additionally, fear of colostomy leaks disrupts religious practices, such as prayer, adding to patients' psychological distress. The absence of community-based programs to educate employers and promote inclusive hiring practices leaves many patients feeling isolated and unsupported in their efforts to reintegrate into the workforce. This highlights the

need for broader societal changes to address stigma and create more inclusive environments for individuals managing chronic health conditions[45]. Additionally, the stigma surrounding cancer and the embarrassment of the procedure create significant psychological barriers to early detection and treatment. Cancer-related taboos and stigma related to the digital rectal exam and colonoscopy are well-documented in studies as a common barrier to care in many cultures, particularly in conservative societies [46, 47].

On the other hand, religious faith and acceptance of one's destiny were vital facilitators. The majority of patients found strength in their religious faith and in coping with the emotional strain of their diagnosis and treatment. Other studies echoed our finding, reiterating that religious beliefs and practices are a powerful coping mechanism for cancer patients [48, 49]. Furthermore, the support from the community NGOs was another facilitator for patients' treatment. The healthcare system in Egypt is multifaceted, involving a wide range of public, private, and non-governmental entities that contribute to the management, financing, and delivery of medical care. There is a strong presence of NGOs and faith-based charities which help facilitate the widespread accessibility to health care and coverage across Egypt in both urban and rural areas[50]. The strong connection between family and community in Egyptian culture significantly influences the country's charitable giving, driving the frequent donation of both money and resources to NGOs. NGOs play a crucial role in cancer care, with a particular focus on prevention, early detection, and patient support. They are pivotal in raising awareness, advocating for cancer prevention, offering screening programs, and supporting treatment[51].

Within the health service organization, our study identified several barriers, including misdiagnoses, lack of proper preparation for diagnostic procedures, insufficient patient education, and lack of structured programs for diet and physical activity. These findings mirror others reporting similar findings. Health literacy, lack of physician recommendation of screening, and misdiagnosis were reported in different studies in the region, leading to advanced-stage diagnoses [18, 39, 52]. Emphasis should be placed on the need for improved physician communication to address patients' concerns and fears related to diagnostic procedures like colonoscopy besides increasing awareness among physicians to suspect CRC at the earliest stage and promoting screening.

Although physicians mentioned the importance of physical activity, and eating well and advised against prolonged bed rest, our study identified a need for more detailed diet and physical plans including referral to a nutritionist and physiotherapist post-CRC diagnosis and treatment, without which, many patients in our study reverted to prior eating habits. Many patients who quit their jobs suffered from poor psychological states from spending most of their time at home, which might explain both the lack of appetite and the decreased level of physical activity. This lack of referral is not unusual in Egypt in that other studies identified malnutrition among CRC patients may worsen health and prevent effective treatments [25, 53, 54]. Implementing nutritional therapy during the treatment of CRC patients in Egypt is essential to enhance patient responses, prevent side effects, and shorten recovery time

[55]. Moreover, patients perceive physical activity as the ability to carry out daily tasks such as going to work, buying dinner, and meeting with friends, and family while only mentioning walking as a form of exercise. This aligns with the results of previous studies that highlighted the lack of physician recommendations regarding what form of physical activity to do and limited access to exercise counseling as major barriers to following physical activity guidelines [56, 57].

At the interpersonal level, the fear of colonoscopy among family and friends impacted patients while, conversely, patients were afraid of peer judgment on colonoscopy and cancer diagnoses. This aligns with findings that highlighted the lack of support from family and friends as a barrier to the uptake of colonoscopy [58]. However, the role of family, friends, and peers was critical in shaping patients' experiences with treatment. Many participants highlighted the emotional and practical support they received from loved ones as a key facilitator in their journey toward treatment and recovery. Knowing someone who survived cancer motivates patients to adhere to treatment and lifestyle recommendations. Social support has been widely recognized in the literature as a crucial determinant of health outcomes, particularly for cancer patients[59].

Individual-level barriers to screening included the lack of awareness and initial perception of their symptoms as minor until serious complications appeared, mainly rectal bleeding and intestinal obstruction. Similar barriers are established in the literature [60-62], highlighting the importance of increasing awareness among the general population about the potential seriousness of CRC symptoms to counteract denial, fatalism, and other coping mechanisms. Furthermore, fear of colonoscopy and perceived fear of pain, lack of privacy, and embarrassment were other factors delaying diagnosis. The primary compelling factor driving voluntarily seeking treatment was deterioration of their condition and fear of death in addition to advice and assurance from their treating physicians. Similar themes were identified in the literature [63-66]. Finally, barriers, such as decreased physical activity, loss of employment, poverty, and physical symptoms from chemotherapy align with other literature reporting similar issues [56, 67]. These findings are consistent with studies that highlight the socioeconomic burdens of cancer care in Egypt where most patients are predominantly living in poverty [68, 69].

The major individual-level facilitator was a common thread of resilience observed among all participants upon receiving their diagnosis. They expressed faith, acceptance of the situation, and a belief in improvement through treatment, encouraging them to continue treatments despite unpleasant side effects. This may be traced to the nature of Arab culture and religions, especially Islam, which includes refraining from expressing negative emotions to others or openly discussing mental health [70, 71]. Cultural taboos about discussing mental health problems is perceived as a defect in faith, which highlights the importance of psychosocial support and counseling services to help patients cope with the emotional impact of receiving a cancer diagnosis.

Finally, the findings of this study highlight the urgent need for culturally adapted policy reforms that consider barriers and facilitators of CRC diagnoses and treatment journeys in Egypt. First, it is crucial to adapt the implementation of CRC screening programs to be inclusive of all people from different backgrounds and rural areas. The absence of preventive measures exacerbates the burden on already strained healthcare systems, increasing the costs of treating advanced cancer cases, which often require more complex and expensive interventions. Health policy reforms should enhance accessibility to screening services and ensure that diagnostic and treatment options are available to all patients, particularly in rural areas where access to healthcare is limited. In 2022, Nawwar et al, developed a theory-based intervention to promote colorectal cancer screening in Egypt using guaiac fecal occult blood test (gFOBT) kits which is cheap, easy to use, and can be distributed across primary care units in Egypt [72]. Following this study, the gFOBT became available as part of the country's 100 Million Healthy Lives Campaign [73] However, the test should be in all primary care units, and healthcare professionals should be trained and required to follow CRC routine screening guidelines. Additionally, access to tests should be free of charge, particularly if people living in limited-resource settings are to be targeted by screening programs. Furthermore, colonoscopies should be covered for positive FOBT results.

Secondly, culturally sensitive education programs are needed to address the stigma and fear surrounding colonoscopy and CRC[74]. These programs should be designed, integrated, and provided in collaboration with local religious and community leaders to ensure that they are culturally appropriate and widely accepted. Additionally, healthcare providers must be trained to offer clear and comprehensive patient education, particularly regarding lifestyle modifications and treatment options. Finally, health service organizations should invest in developing structured support programs for diet and physical activity guidance, to improve patient outcomes and quality of life. Collaboration between government institutions and local healthcare providers is crucial for ensuring that these programs are sustainable and accessible to all. Additionally, further research into gender-specific barriers, such as the unique challenges faced by women in accessing CRC care, would provide valuable insights for developing targeted interventions.

Strengths and Limitations

To the authors' knowledge, this is the first qualitative study to explore CRC patient's journey in Egypt. Our study is distinguished by its method of performing in-person interviews with patients, which gave us valuable insights into their experiences and needs. It also addressed an important gap in knowledge about the role of diet and physical activity in Egyptian colorectal cancer patients and shed light on the barriers and facilitators to adopting a better lifestyle during and after cancer treatment.

On the other hand, this study has limitations to note. We used a convenience sample from one center which may limit the generalizability of the findings, especially to communities with higher income levels. Additionally, semi-structured interviews rely on participants' subjective experiences and emotions.

Unlike anonymous questionnaires, patients in in-person interviews may tend to express more positive aspects of their experience than negative ones; however, we tried to address that by providing questions and repeating them in different formats to obtain more comprehensive and clear answers. Throughout the study, dominance, and recalcitrance did not appear to be problematic.

Conclusions

This study provides valuable insights into the lived experiences of CRC patients in Egypt. Using the SEM as a framework, our findings highlight significant barriers, including limited awareness, fear of procedures, financial constraints, and healthcare accessibility issues, while emphasizing the role of social support from family and friends, faith resilience, and free healthcare at university hospitals as facilitators. This study underscores the urgent need for culturally adapted policy reforms to improve CRC care in Egypt, including expanding CRC screening programs, integrating routine diagnostic protocols, and ensuring affordability, particularly for underserved populations. It reveals a pressing need for improved healthcare communication and structured support systems, including dietary and physical activity plans, to enhance patient outcomes post-diagnosis. Future research should focus on gender-specific barriers and long-term psychosocial impacts to inform tailored culturally sensitive interventions and enhance patient outcomes. By leveraging the insights gained from our study, healthcare providers and policymakers can work towards improving the overall health outcomes for CRC patients in Egypt, ultimately leading to better survival rates and quality of life for patients living with CRC.

Abbreviations

Declarations

Ethical approval

The study was conducted in accordance with the Declaration of Helsinki and approved by the Ethics Committee of the Faculty of Medicine, Alexandria University, Egypt (I.R.B. number: 00012098, date of approval 7/07/2023).

Consent to participate

Informed consent to participate in the study was obtained from all participants.

Availability of data and materials

The interview records and data used and/or analyzed during the current study are available from the corresponding author upon reasonable request.

Competing interests

The authors declare that they have no competing interests.

Authors' contributions: All authors contributed equally to this work.

Funding: This study was supported by an institutional grant from the UAB Mary Heersink Institute of Global Health at the University of Alabama at Birmingham, Birmingham, AL, USA.

References:

1. Xi, Y. and P. Xu, *Global colorectal cancer burden in 2020 and projections to 2040.* Transl Oncol, 2021. **14**(10): p. 101174.
2. Sung, H., et al., *Global Cancer Statistics 2020: GLOBOCAN Estimates of Incidence and Mortality Worldwide for 36 Cancers in 185 Countries.* CA Cancer J Clin, 2021. **71**(3): p. 209-249.
3. Arnold, M., et al., *Global patterns and trends in colorectal cancer incidence and mortality.* Gut, 2017. **66**(4): p. 683-691.
4. Murphy, N., et al., *Lifestyle and dietary environmental factors in colorectal cancer susceptibility.* Mol Aspects Med, 2019. **69**: p. 2-9.
5. Siegel, R.L., et al., *Colorectal cancer statistics, 2020.* CA Cancer J Clin, 2020. **70**(3): p. 145-164.
6. Brenner, H. and C. Chen, *The colorectal cancer epidemic: challenges and opportunities for primary, secondary and tertiary prevention.* Br J Cancer, 2018. **119**(7): p. 785-792.
7. Ferlay, J., et al., *Global cancer observatory: cancer today.* Lyon: International agency for research on cancer, 2020. **20182020**.
8. World Health Organization, G.C.O. *WHO EAST MEDITERRANEAN*

REGION (EMRO) CANCER. 2022 [cited 2024; Available from: https://gco.iarc.who.int/media/globocan/factsheets/populations/993-who-east-mediterranean-emro-fact-sheet.pdf.
9. Al Zaabi, A., et al., *Early Onset Colorectal Cancer in Arabs, Are We Dealing with a Distinct Disease?* Cancers (Basel), 2023. **15**(3).
10. Nikbakht, H.A., et al., *Survival Rate of Colorectal Cancer in Eastern Mediterranean Region Countries: A Systematic Review and Meta-Analysis.* Cancer Control, 2020. **27**(1): p. 1073274820964146.

11. World Health Organization, G.C.O., World Health Organization, Global Cancer Observatory, . *EGYPT CANCER FACT SHEET*. 2022 [cited 2024; Available from: https://gco.iarc.who.int/media/globocan/factsheets/populations/818-egypt-fact-sheet.pdf.
12. Rashad, N., et al., *Early-Onset Colorectal Cancer in Egypt: Pathological Characters, Patterns of Care, and Survival Compared to Average-Age Onset Colorectal Cancer: A Retrospective Multicenter Study.* JCO Global Oncology, 2024(10): p. e2300372.
13. Elhadidy, A.A. and T. Haydara, *Increase Young Age Incidence of Colorectal Carcinoma Among Cohort of Egyptian Population.* Al-Azhar International Medical Journal, 2022. **3**(1): p. 71-75.
14. Akimoto, N., et al., *Rising incidence of early-onset colorectal cancer - a call to action.* Nat Rev Clin Oncol, 2021. **18**(4): p. 230-243.
15. Siegel, R.L., et al., *Colorectal cancer statistics, 2017.* CA Cancer J Clin, 2017. **67**(3): p. 177-193.
16. Metwally, I.H., et al., *Epidemiology and survival of colon cancer among Egyptians: a retrospective study.* Journal of Coloproctology, 2021. **38**(01): p. 024-029.
17. Pan, J., et al., *Colonoscopy Reduces Colorectal Cancer Incidence and Mortality in Patients With Non-Malignant Findings: A Meta-Analysis.* Official journal of the American College of Gastroenterology | ACG, 2016. **111**(3).
18. Allam, A.R., et al., *Colonoscopy screening for colorectal cancer in Egypt: a nationwide cross-sectional study.* BMC Cancer, 2024. **24**(1): p. 131.
19. Aicr and Wcrf, *Diet, nutrition, physical activity and colorectal cancer.*
20. Rock, C.L., et al., *American Cancer Society guideline for diet and physical activity for cancer prevention.* CA Cancer J Clin, 2020. **70**(4): p. 245-271.
21. Song, M., et al., *Fiber Intake and Survival After Colorectal Cancer Diagnosis.* JAMA Oncol, 2018. **4**(1): p. 71-79.
22. Meyerhardt, J.A., et al., *Physical Activity and Survival After Colorectal Cancer Diagnosis.* Journal of Clinical Oncology, 2006. **24**(22): p. 3527-3534.
23. Van Blarigan, E.L. and J.A. Meyerhardt, *Role of physical activity and diet after colorectal cancer diagnosis.* J Clin Oncol, 2015. **33**(16): p. 1825-34.
24. Ricci, C., et al., *Diet and sedentary behaviour in relation to cancer survival. A report from the national health and nutrition examination survey linked to the U.S. mortality registry.* Clin Nutr, 2020. **39**(11): p. 3489-3496.
25. El-Kassas, D., et al., *The Potential of Malnutrition among Colorectal Cancer Adult Patients; Case-Control Study.* Journal of Home Economics-Menofia University, 2022. **32**(4): p. 81-100.
26. Mahfouz, E.M., et al., *The role of dietary and lifestyle factors in the development of colorectal cancer: case control study in Minia, Egypt.* Cent Eur J Public Health, 2014. **22**(4): p. 215-222.
27. Aly, S.M., et al., *Physical Activity among Colorectal Cancer Patients and Survivors in Egypt.* Asian Pac J Cancer Prev, 2023. **24**(9): p. 3117-3123.
28. Khalifa, S.M., et al., *Psychological Assessment and Quality of Life of patients with Colorectal Cancer at Assiut University Hospital and South Egypt Cancer Institute.* Assiut Scientific Nursing Journal, 2014. **2**(4): p. 22-35.
29. Salika, T., et al., *Associations between diagnostic pathways and care experience in colorectal cancer: evidence from patient-reported data.* Frontline Gastroenterol, 2018. **9**(3): p. 241-248.
30. Oliver, A. and C.C. Greenberg, *Measuring outcomes in oncology treatment: the importance of patient-centered outcomes.* Surg Clin North Am, 2009. **89**(1): p. 17-25, vii.

31. Kilanowski, J.F.P.R.A.C.F., *Breadth of the Socio-Ecological Model.* J Agromedicine, 2017. **22**(4): p. 295-297.
32. Centers for Disease Control and Prevention and ATSDR. *The Social-Ecological Model: A framework for prevention*. n.d. January 28, 2020 August 27, 2020]; Available from: https://www.atsdr.cdc.gov/community-engagement/php/chapter-1/models-frameworks.html?CDC_AAref_Val=https://www.atsdr.cdc.gov/communityengagement/pce_models.html.
33. International, Q., *NVivo Qualitative Data Analysis Software*. 2015, QSR International Pty Ltd.
34. McLeroy, K.R., et al., *An ecological perspective on health promotion programs.* Health education quarterly, 1988. **15**(4): p. 351-377.
35. Mounir, A., et al., *Epidemiology of Colorectal Cancer, Incidence, Survival, and Risk Factors: Cairo University Center of Oncology and Nuclear Medicine Experience.* The Egyptian Journal of Hospital Medicine, 2022. **89**(2): p. 7061-7070.
36. Metwally, I.H., et al., *Epidemiology and survival of colon cancer among Egyptians: a retrospective study.* Journal of Coloproctology (Rio de Janeiro), 2018. **38**(1): p. 24-29.
37. Organization, W.H., *Increasing access to health workers in remote and rural areas through improved retention: global policy recommendations*. 2010: World Health Organization.
38. Martinez, M.E., et al., *Cancer Screening in Low- and Middle-Income Countries.* American Society of Clinical Oncology Educational Book, 2024. **44**(3): p. e431272.
39. Brand Bateman, L., et al., *Designing an Effective Colorectal Cancer Screening Program in Egypt: A Qualitative Study of Perceptions of Egyptian Primary Care Physicians and Specialists.* Oncologist, 2020. **25**(10): p. e1525-e1531.
40. Castelo, M., et al., *Time to diagnosis and treatment in younger adults with colorectal cancer: A systematic review.* Plos one, 2022. **17**(9): p. e0273396.
41. Weller, D., et al., *Diagnostic routes and time intervals for patients with colorectal cancer in 10 international jurisdictions; findings from a cross-sectional study from the International Cancer Benchmarking Partnership (ICBP).* BMJ open, 2018. **8**(11): p. e023870.
42. Vega, P., F. Valentin, and J. Cubiella, *Colorectal cancer diagnosis: Pitfalls and opportunities.* World journal of gastrointestinal oncology, 2015. **7**(12): p. 422.
43. Fasseeh, A., et al., *Healthcare financing in Egypt: a systematic literature review.* J Egypt Public Health Assoc, 2022. **97**(1): p. 1.
44. World Bank. *Poverty headcount ratio at national poverty lines (% of population) - Egypt, Arab Rep.* 2023 [cited 2024; Available from: https://data.worldbank.org/indicator/SI.POV.NAHC?locations=EG.
45. Stergiou-Kita, M., C. Pritlove, and B. Kirsh, *The "Big C"-stigma, cancer, and workplace discrimination.* J Cancer Surviv, 2016. **10**(6): p. 1035-1050.
46. Goldman, R.E., J.A. Diaz, and I. Kim, *Perspectives of colorectal cancer risk and screening among Dominicans and Puerto Ricans: stigma and misperceptions.* Qual Health Res, 2009. **19**(11): p. 1559-68.
47. Unger-Saldaña, K., et al., *Barriers and facilitators for colorectal cancer screening in a low-income urban community in Mexico City.* Implementation Science Communications, 2020. **1**(1): p. 64.
48. Benidir, A., M.J. Levert, and K. Bilodeau, *The Role of Islamic Beliefs in Facilitating Acceptance of Cancer Diagnosis.* Curr Oncol, 2023. **30**(9): p. 7789-7801.

49. Balboni, T.A., et al., *Religiousness and spiritual support among advanced cancer patients and associations with end-of-life treatment preferences and quality of life.* J Clin Oncol, 2007. **25**(5): p. 555-60.
50. Elshamy, K., *Challenges and future trends for cancer care in Egypt.* Cancer Care in Countries and Societies in Transition: Individualized Care in Focus, 2016: p. 117-146.
51. Ibrahim, A.H. and E. Shash, *General Oncology Care in Egypt*, in *Cancer in the Arab World*, H.O. Al-Shamsi, et al., Editors. 2022, Springer Singapore: Singapore. p. 41-61.
52. Alduraywish, S.A., et al., *Barriers of colorectal cancer screening test among adults in the Saudi Population: A Cross-Sectional study.* Preventive Medicine Reports, 2020. **20**: p. 101235.
53. Abdelfatah, D., et al., *Nutritional Assessment of Egyptian Adults with Hepatic and Gastrointestinal Malignancies: Cross sectional study.* The Egyptian Journal of Community Medicine, 2022. **40**(2): p. 134-142.
54. Abd Allah, E.S., H.M.M. Gad, and H.R. Abdel-Aziz, *Nutritional status and its contributing factors among older adults with cancer receiving chemotherapy.* Clinical Nursing Research, 2020. **29**(8): p. 650-658.
55. Lewandowska, A., et al. *Nutritional Treatment of Patients with Colorectal Cancer*. International Journal of Environmental Research and Public Health, 2022. **19**, DOI: 10.3390/ijerph19116881.
56. Clifford, B.K., et al., *Barriers and facilitators of exercise experienced by cancer survivors: a mixed methods systematic review.* Supportive Care in Cancer, 2018. **26**: p. 685-700.
57. Smith, L., et al., *Cancer survivors' attitudes towards and knowledge of physical activity, sources of information, and barriers and facilitators of engagement: A qualitative study.* Eur J Cancer Care (Engl), 2017. **26**(4).
58. Kerrison, R.S., et al., *Barriers and facilitators to colonoscopy following fecal immunochemical test screening for colorectal cancer: A key informant interview study.* Patient Educ Couns, 2022. **105**(6): p. 1652-1662.
59. Haviland, J., et al., *Social support following diagnosis and treatment for colorectal cancer and associations with health-related quality of life: Results from the UK ColoREctal Wellbeing (CREW) cohort study.* Psycho-oncology, 2017. **26**(12): p. 2276-2284.
60. Leal, I.M., et al., *Understanding patients' experiences of diagnosis and treatment of advanced colorectal cancer in a safety-net hospital system: A qualitative study.* Diseases of the Colon & Rectum, 2018. **61**(4): p. 504-513.
61. Oberoi, D., et al., *Help-seeking experiences of men diagnosed with colorectal cancer: a qualitative study.* European Journal of Cancer Care, 2016. **25**(1): p. 27-37.
62. van Deursen, L., et al., *Exploring colorectal cancer survivors' perspectives on improving care delivery and the role of e-health technology: a qualitative study.* Supportive Care in Cancer, 2023. **31**(9): p. 544.
63. Bertels, L., et al., *Motives for non-adherence to colonoscopy advice after a positive colorectal cancer screening test result: a qualitative study.* Scandinavian journal of primary health care, 2020. **38**(4): p. 487-498.
64. Kerrison, R.S., et al., *Barriers and facilitators to colonoscopy following fecal immunochemical test screening for colorectal cancer: A key informant interview study.* Patient Education and Counseling, 2022. **105**(6): p. 1652-1662.
65. Kerrison, R.S., et al., *Barriers to colonoscopy in UK colorectal cancer screening programmes: Qualitative interviews with ethnic minority groups.* Psycho-Oncology, 2023. **32**(5): p. 779-792.

66. Bie, A.K.L. and J. Brodersen, *Why do some participants in colorectal cancer screening choose not to undergo colonoscopy following a positive test result? A qualitative study.* Scandinavian journal of primary health care, 2018. **36**(3): p. 262-271.
67. Viscuse, P.V., et al., *Integrative medicine in cancer survivors.* Current opinion in oncology, 2017. **29**(4): p. 235-242.
68. Gany, F., et al., *Financial and food security challenges of Egyptian women undergoing breast cancer treatment.* Support Care Cancer, 2020. **28**(12): p. 5787-5794.
69. Hofmarcher, T., et al., *The Disease Burden and Economic Burden of Cancer in 9 Countries in the Middle East and Africa.* Value in Health Regional Issues, 2023. **37**: p. 81-87.
70. Saunders, C.H., et al., *'Because Everybody is so Different': a qualitative analysis of the lived experiences and information needs of rectal cancer survivors.* BMJ open, 2021. **11**(5): p. e043245.
71. González-Herrera, A., et al., *Colorectal cancer: a qualitative study of coping strategies used by survivors, with associated social determinants.* Archives of Public Health, 2023. **81**(1): p. 111.
72. Nawwar, A., et al., *Using Intervention Mapping to Develop a Theory-Based Intervention to Promote Colorectal Cancer Screening in Egypt.* Asian Pac J Cancer Prev, 2022. **23**(6): p. 1975-1981.
73. Egypt State Information Service. *1st phase of presidential initiative on detection of cancer*. 2023 [cited 2024; Available from: https://sis.gov.eg/Story/182055/1st-phase-of-presidential-initiative-on-detection-of-cancer-kicks-off?lang=en-us.
74. Manne, S.L., et al., *Culturally-adapted behavioral intervention to improve colorectal cancer screening uptake among foreign-born South Asians in New Jersey: the Desi Sehat trial.* Ethnicity & health, 2021. **26**(4): p. 554-570.

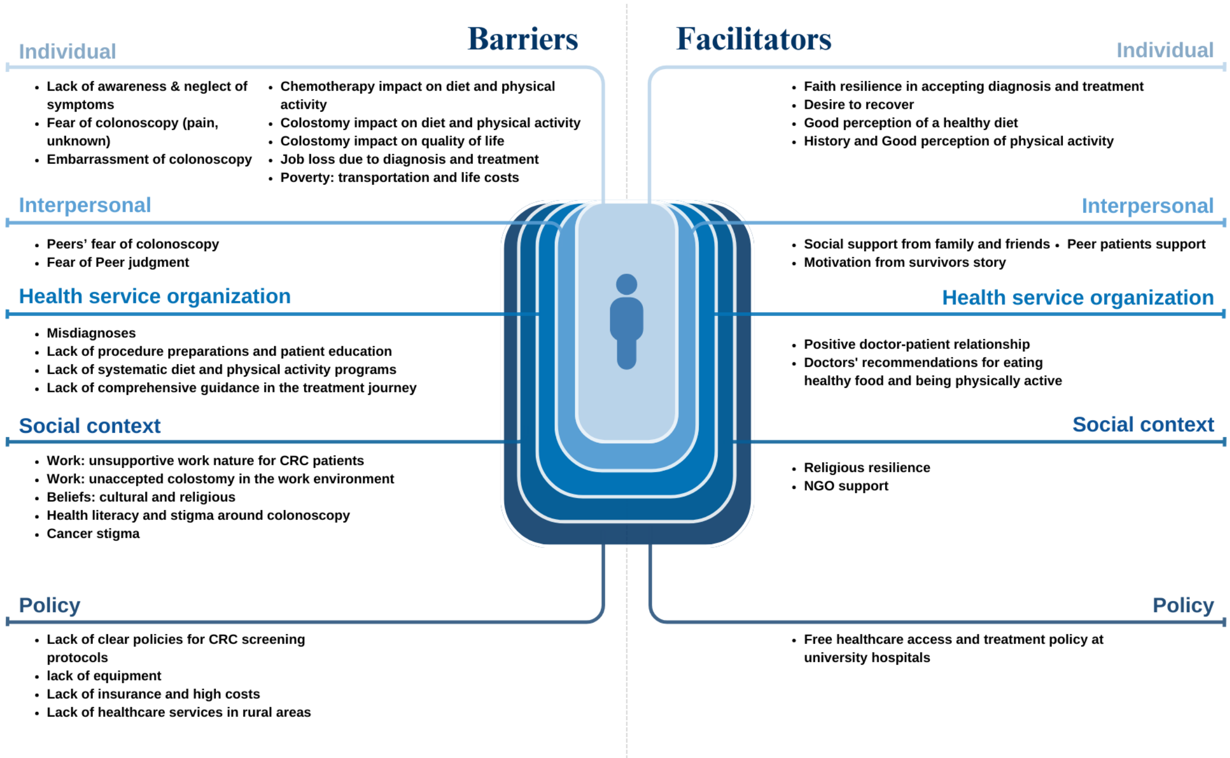

Figure 1. Perceived Barriers and Facilitators at the different levels of the SEM

Click here to access/download
Supporting Information
Ethics 2023 En.pdf
