# Supplementary material for: Mapping the colorectal cancer patient journey in Egypt: A qualitative study of diagnosis, treatment, and lifestyle perspectives
Source: PLoS One. 2025 Jul 2;20(7):e0326144. doi: 10.1371/journal.pone.0326144 (PMC12220998; doi:10.1371/journal.pone.0326144)
Supplement: Attachment — Submitted filename: Reviewers Comments C-CREP Plose One lbb.docx [file pone.0326144.s007.docx]

|  | Comments | Respond |
| --- | --- | --- |
|  | ***Editor Comments*** | |
| 1- | It is advisable to emphasize redrafting the current manuscript to make it more concise, clear, and organized for easy readability by the audience.  Advise redrafting a few sections of the paper to make it more concise keeping only the relevant information. Some of the sections where this strategy can be applied are:-  3.1.1  3.1.3- last 3 paragraphs  3.1.4 - last paragraph | Thank you for your valuable feedback. We have carefully reviewed the manuscript and revised the specified sections (3.1.1, 3.1.3, 3.1.4) to improve clarity, conciseness, and overall readability. In addition, we applied the same redrafting strategy to other sections of the results that appeared lengthy or repetitive, while ensuring that key participant insights and thematic content were preserved. We appreciate your guidance in helping us strengthen the structure and presentation of the manuscript. |
| 2- | Advise to use Table 2 as a supplemental note as there is duplication of the patient’s quotations throughout the text. | Thanks for the suggestion! We moved Table 2 to the supplemental note to avoid duplication of patient quotes in the text. |
| 3- | Discussion:  The discussion section should be restructured as this section is very long and difficult to follow. Advise to categorize the codes and themes in two different categories as mentioned in the supplemental: Perceived barriers and Perceived facilitators. Under those headings advise to provide key findings of the study. For eg., the authors may choose to categorize the findings under different headings:-  Screening and diagnosis: stigma to do a colonoscopy, health care organization, health care policy, individual factors, social factors  Management: access to care, job safety, diet, physical therapy, mental health  Post-treatment: unemployment, social stigma | Thank you for your helpful recommendation regarding the structure of the Discussion section. In response, we have **reorganized the Discussion into two main categories: *Perceived Barriers* and *Perceived Facilitators***, as suggested. Under each category, we now present the key findings in alignment with the patient journey—covering diagnosis, treatment, and survivorship phases. This revised structure improves clarity and flow, allowing readers to more easily follow the thematic progression of the results. |
| 4- | Provide strategies to improve the screening, diagnosis, and management of CRC in Egypt based on the findings of the study.  Some suggestions are:-             Screening:  Involve religious leaders and organizations to create awareness about the condition  Involve local media or social groups to create awareness  Involve NGOs in social awareness  More training during medical school about the condition  Special focus on targeting the younger population because of ‘Early onset CRC’ in this population.              Diagnosis and management:  Government support for cancer survivors  Involving NGOs to help people navigate the journey of screening, access to care  Peer support groups for those undergoing cancer treatment and post-cancer treatment. | Thank you for your valuable feedback. In response, we would like to confirm that we have already expanded our **Recommendations and Implications** section to address the full spectrum of barriers identified in the study, providing **specific, actionable strategies** to improve CRC screening, diagnosis, and management in Egypt.  Our recommendations include:   - **Implementation of culturally adapted, accessible screening programs**, particularly in underserved areas. - **Training healthcare providers and medical students** to identify early CRC symptoms, including early-onset cases, and follow national screening guidelines. - **Collaboration with religious leaders, NGOs, and local media** to raise public awareness, reduce stigma, and promote early detection. - **Enhanced patient education** and **structured care pathways** covering diet, physical activity, and psychosocial support. - **Establishment of peer support groups**, and **NGO involvement** to assist patients in navigating care and accessing resources. - **Policy-level recommendations** for financial support, employment reintegration, and survivorship care programs.   These align closely with your suggestions, and we appreciate the opportunity to emphasize their inclusion in the manuscript. |
| 5- | Please upload a completed version of your questionnaire as Supporting Information when you resubmit your manuscript. | Thank you for the request. I will upload the completed version of the questionnaire as Supporting Information when resubmitting the manuscript. |
| 6- | We note that the grant information you provided in the ‘Funding Information’ and ‘Financial Disclosure’ sections do not match. When you resubmit, please ensure that you provide the correct grant numbers for the awards you received for your study in the ‘Funding Information’ section. | Thank you for your comment. We apologize for the confusion. This study was supported by an institutional grant from the UAB Mary Heersink Institute of Global Health at the University of Alabama at Birmingham, Birmingham, AL, USA. However, there is no specific grant number associated with this funding. |
| 7- | Thank you for stating the following financial disclosure: “This study was supported by an institutional grant from the UAB Mary Heersink Institute of Global Health at the University of Alabama at Birmingham, Birmingham, AL, USA.”  Please state what role the funders took in the study. If the funders had no role, please state: "The funders had no role in study design, data collection and analysis, decision to publish, or preparation of the manuscript." If this statement is not correct you must amend it as needed. Please include this amended Role of Funder statement in your cover letter; we will change the online submission form on your behalf. | Thank you for your comment. We added the Role of Funder statement to the cover letter as follows: "The funders had no role in study design, data collection and analysis, decision to publish, or preparation of the manuscript." |
| 8- | In the online submission form, you indicated that “The interview records and data used and/or analyzed during the current study are available from the corresponding author upon reasonable request.”  All PLOS journals now require all data underlying the findings described in their manuscript to be freely available to other researchers, either 1. In a public repository, 2. Within the manuscript itself, or 3. Uploaded as supplementary information. This policy applies to all data except where public deposition would breach compliance with the protocol approved by your research ethics board. If your data cannot be made publicly available for ethical or legal reasons (e.g., public availability would compromise patient privacy), please explain your reasons on resubmission and your exemption request will be escalated for approval. | The interview records and data used and/or analyzed during the current study cannot be shared publicly to protect participant confidentiality and privacy. Excerpts of data relevant to the study’s findings are available from the corresponding author upon reasonable request |
| 9- | Please see the attached PDF for further comments (marked and comments annoted). | Thank you we reviewed it. |
| 10- | Your ethics statement should only appear in the Methods section of your manuscript. If your ethics statement is written in any section besides the Methods, please move it to the Methods section and delete it from any other section. Please ensure that your ethics statement is included in your manuscript, as the ethics statement entered into the online submission form will not be published alongside your manuscript. | Thank you for your guidance. We have revised the manuscript to ensure that the full ethics statement appears only in the *Methods* section, under a newly added subsection titled *2.5. Ethical Considerations and Approval*. The statement now reads as follows:  *“The protocol for this study was approved by the Ethics Committee of the Faculty of Medicine, Alexandria University, Egypt (I.R.B. number: 00012098, date of approval 9 July 2023). The study was conducted in accordance with the ethical standards of the Declaration of Helsinki and ethical guidelines [33]. All participants were informed that their participation was voluntary, and written informed consent was obtained from all participants prior to the interviews. Participants were provided with an Information Sheet detailing the study objectives, procedures, and their rights. Interview recordings and demographic questionnaire responses were securely stored on a password-protected computer accessible only to the lead investigator to ensure data confidentiality.”* |
| 11- | Please include a separate caption for each figure in your manuscript. | Thank you. We made sure the caption is included |
|  | Please include captions for your Supporting Information files at the end of your manuscript, and update any in-text citations to match accordingly. Please see our Supporting Information guidelines for more information: [http://journals.plos.org/plosone/s/supporting-information](http://track.editorialmanager.com/CL0/http:%2F%2Fjournals.plos.org%2Fplosone%2Fs%2Fsupporting-information/1/010f01963267dea6-59971e69-9afa-4a8b-aa2d-c0dcfe0d0b27-000000/OWvqOvQoBPmHBbDBPx-FbAeNq0N6kjFQmdfxASYSnZs=206). | Thank you for the note. We have added captions for all Supporting Information files at the end of the manuscript and updated the in-text citations to match accordingly, following the journal’s guidelines. |
|  | ***Reviewer Comments*** | |
| 12- | Reviewer #1: In this manuscript, the authors provide insights into the real-life experiences of CRC patients in Egypt. Results were very important to improve CRC diagnosis and treatment outcomes. Besides that, I found several concerns I described below. -The manuscript is too long. Authors must summarize some sections and avoid repetition. -Recruitment Period was too brief. Can authors justify this? In my opinion, the number of patient was very limited (N= 19) and this is not enough for statistical validation. | Thank you for your valuable comments and suggestions. We appreciate your recognition of the importance of the study’s findings and have addressed your concerns as follows:   1. **Manuscript Length and Repetition:**  We have carefully reviewed the manuscript and revised multiple sections to improve clarity and reduce repetition. The **Results** and **Discussion** sections were restructured to be more concise and better organized under the headings *Perceived Barriers* and *Perceived Facilitators*, as recommended by the editorial team. 2. **Recruitment Period and Sample Size:**  The recruitment period (August 1 to September 30, 2023) was appropriate for our study design, as it was based on the availability of eligible, consenting patients during that time. The **sample size of 19 participants** was determined using the principle of **thematic saturation**, which is a recognized standard in qualitative research. Saturation was defined as the point at which no new themes or insights were emerging from the interviews.  We have clarified this point in the **Methods** section. 3. **Statistical Validation Concern:**  As this is a **qualitative study**, the aim was not statistical generalization but rather an in-depth understanding of patients’ experiences. Accordingly, the term *“Statistical Analysis”* has been revised to *“Data Analysis”* in the manuscript to better reflect the qualitative methodology and the use of thematic content analysis. |
| 13- | Reviewer #2: Dear authors, thank you for this intersting topic here are my comments for more clarity: #It is bettet yo replace “Statistical Analysis” with “Data Analysis” since this is qualitative and add a brief explanation of how thematic saturation was assessed (e.g., “coding continued until no new themes emerged”). #Table 1: please correct inconsistencies (e.g., text says “12 males, 7 females” but Table 1 says “17 males”; age mean in text is 53.4 but Table 1 says 54.8). Standardize BMI categories (e.g., “Optimum range” could be “Normal” per WHO). | Thank you for your thoughtful review and helpful comments. We appreciate your time and valuable suggestions, which have improved the clarity and accuracy of our manuscript.   1. **Title correction:** We have replaced “Statistical Analysis” with “Data Analysis” to reflect the qualitative nature of the study. Additionally, we have added a brief explanation of how thematic saturation was assessed, stating that *"Data coding continued until thematic saturation was reached, defined as the point at which no new themes or insights were identified."* 2. **Table 1 inconsistencies:** We have corrected the discrepancies in the text with Table 1 regarding the number of participants by sex and the reported mean age. In the study, we have 12 males and 7 females with a mean age of 54.8 ±10.2. 3. **BMI category standardization:** We have revised the BMI categories in Table 1 to align with the World Health Organization (WHO) classification. Specifically, the term “Optimum range” has been replaced with “Normal weight.” |
